# The role of BATF in immune cell differentiation and autoimmune diseases

**DOI:** 10.1186/s40364-025-00733-x

**Published:** 2025-01-29

**Authors:** Xiaomeng Wang, Yue Hong, Jinmei Zou, Bo Zhu, Chao Jiang, Liwei Lu, Jie Tian, Jing Yang, Ke Rui

**Affiliations:** 1 Department of Laboratory Medicine, Institute of Medical Immunology, Affiliated Hospital of Jiangsu University, Zhenjiang, China; 2 Department of Immunology, Jiangsu Key Laboratory of Laboratory Medicine, School of Medicine, Jiangsu University, Zhenjiang, China; 3 Department of Hematology and Oncology, Children’s Hospital of Nanjing Medical University, Nanjing, China; 4 Department of Rheumatology, School of Medicine, Mianyang Central Hospital, University of Electronic Science and Technology of China, Mianyang, China; 5 Department of Orthopaedics, Affiliated Hospital of Jiangsu University, Zhenjiang, China; 6 Department of Pathology, Shenzhen Institute of Research and Innovation, The University of Hong Kong, Hong Kong, China

**Keywords:** BATF, Immune cell, Autoimmune disease, Immunomodulation, Autoimmunity

## Abstract

As a member of the Activator Protein-1 (AP-1) transcription factor family, the Basic Leucine Zipper Transcription Factor (BATF) mediates multiple biological functions of immune cells through its involvement in protein interactions and binding to DNA. Recent studies have demonstrated that BATF not only plays pivotal roles in innate and adaptive immune responses but also acts as a crucial factor in the differentiation and function of various immune cells. Lines of evidence indicate that BATF is associated with the onset and progression of allergic diseases, graft-versus-host disease, tumors, and autoimmune diseases. This review summarizes the roles of BATF in the development and function of innate and adaptive immune cells, as well as its immunoregulatory effects in the development of autoimmune diseases, which may enhance the current understanding of the pathogenesis of autoimmune diseases and facilitate the development of new therapeutic strategies.

## Introduction

The basic leucine zipper transcription factor (BATF) family, which includes BATF, BATF2, and BATF3, is a member of the AP-1 transcription factor family [1]. All BATF family members possess a basic leucine zipper (bZIP) and a DNA-binding domain (DBD), which are primarily involved in mediating protein-protein interactions and DNA binding [2, 3]. The BATF family plays a crucial role in the differentiation and functional regulation of immune cells [1]. BATF expression varies across different cell types; for instance, BATF expression in T cells influences their differentiation and effector functions and is essential for the differentiation of IL-17-producing T helper (Th17) and follicular T helper (Tfh) cells [4, 5]. In the early stages of antiviral and antitumor immune responses, BATF is critically involved in the differentiation of CD8^+^T cells. Additionally, B cells in mice with BATF deletion lack the ability to switch antibody classes [6–8].

Recent studies have shown that BATF is associated with the development of various diseases, including allergic respiratory diseases and antitumor immune responses [1, 9]. Compelling evidence suggests that BATF is involved in the development of multiple sclerosis (MS), rheumatoid arthritis (RA), systemic lupus erythematosus (SLE), experimental autoimmune uveitis (EAU), experimental autoimmune encephalomyelitis (EAE), and inflammatory bowel disease (IBD) [4, 10, 11]. Dysregulated BATF expression may lead to impaired immune suppression in these diseases; for instance, inflammatory responses are significantly reduced in IBD, EAE, and RA mouse models lacking BATF [4, 11, 12]. BATF contributes to the progression of autoimmune diseases by regulating the differentiation and function of Th17, Tfh, Treg, and B cells [13, 14]. Although much less is known about the other family members, BATF2 and BATF3, it is clear that BATF2 is expressed in M1 macrophages and Th1 cells, while BATF3 is mainly expressed in dendritic cells, T cells, and B cells [15, 16]. However, the BATF-mediated molecular pathways involved in autoimmune diseases are only partially elucidated.

In this review, we summarize the role of BATF, a key member of the basic leucine zipper transcription factor family, in regulating immune cell differentiation and function. Further investigation into the structure and function of BATF, as well as its role in autoimmune disease development, may facilitate the identification of potential therapeutic targets for the treatment of autoimmune diseases.

## BATF family and functions

### Expression of BATF

**Fig. 1:**
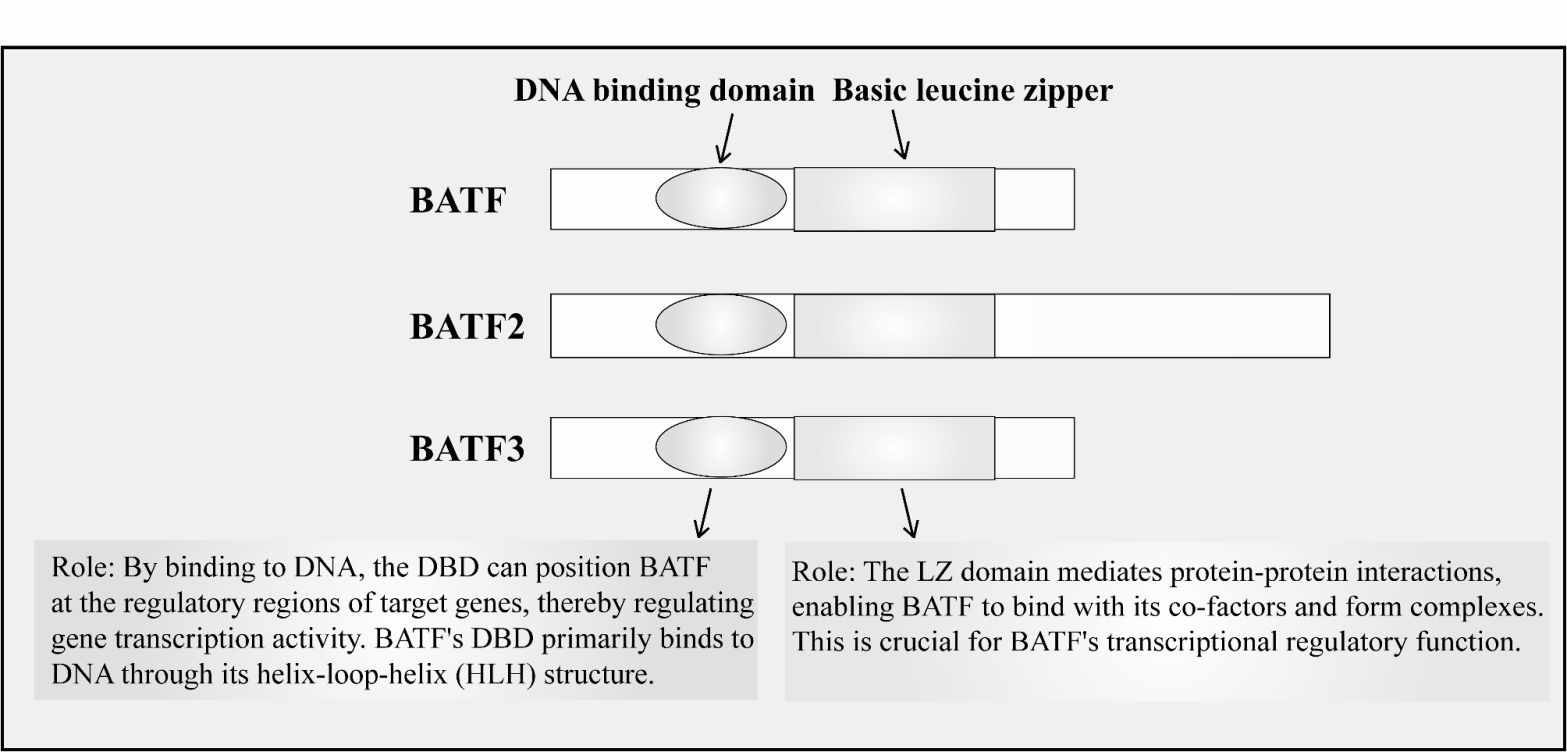
Members of the BATF family possess both a bZIP and a DBD. The leucine zipper is a specific α-helix structure containing repeated leucine residues, typically appearing every seven amino acids. This structure allows BATF to form dimers or multimers with other AP-1 family members (such as c-Jun and c-Fos), thereby enhancing its stability and function. The DBD is responsible for recognizing and binding to specific DNA sequences, usually located in the promoter or enhancer regions of target genes. This binding is specific, depending on the structure of the DBD and the sequence of the DNA

BATF was initially isolated from a gene library of human B cells stimulated by the Epstein-Barr virus (EBV) [17, 18]. An early study reported that BATF expression in human B cells significantly increased after infection with EBV, indicating its involvement in the early response to viral infection. It was also found that telomerase reverse transcriptase (TERT) negatively affects the expression of BZLF1, a key regulatory factor in the EBV lytic cycle, through the NOTCH2/BATF pathway, thus inhibiting the EBV lytic program and maintaining the virus in a latent state [19, 20]. The BATF gene is located on human chromosome 14q24 and consists of three exons. It encodes 304 nucleotides, including the start codon and a complete DBD, and 530 nucleotides spanning the first leucine of the bZIP and other C-terminal residues [21] (Fig. 1). Unlike other members of the AP-1 family, BATF lacks an additional transactivation domain [3, 22]. However, its bZIP domain can form homodimers or heterodimers with other AP-1 factors, such as Jun/Fos, and by binding to TRE (12-O-tetradecanoylphorbol-13-acetate-response element) or CRE (cAMP response elements), it competitively inhibits the activity of Jun/Fos dimers, thus regulating the expression of target genes [22–24]. Additionally, BATF2 has an additional C-terminal structure similar to Fos, but its function remains unclear [3].

BATF, BATF2, and BATF3 are distinct transcription factors within the AP-1 transcription factor family, each exhibiting unique expression patterns and functions within the immune system [3]. These factors are primarily expressed in various immune cells and their precursors, including hematopoietic stem cells, lymphoid cells, and myeloid cells [25–27]. BATF is induced in hematopoietic stem cells by G-CSF/STAT3 signaling, which primarily limits cell renewal and promotes lymphoid differentiation in response to DNA damage [27]. In CD4^+^T cells, BATF is activated by IL-6/STAT3 signaling, promoting the differentiation of Th17 and Tfh cells [4, 5]. It has also been reported that BATF can promote the differentiation of Th9 cells and the production of IL-9 through the TLA1/STAT6 signaling pathway [28, 29]. It interacts with transcription factors such as RORγT, Bcl6, c-Maf, PU.1, and IRF4 to regulate the production of inflammatory cytokines like IL-17, IL-21, IL-22, and IL-9, thus impacting immune balance and inflammatory responses [30, 31]. Additionally, BATF plays a crucial role in Treg cell differentiation and function by interacting with Foxp3 and modulating the production of inhibitory cytokines, thereby affecting their immunoregulatory function [32, 33]. BATF is also indispensable in the differentiation and development of effector CD8^+^T cells, interacting with IRF4 and T-bet to regulate their differentiation [7]. BATF3 promotes the proliferation of CD8^+^T cells and their differentiation into memory cells [34, 35]. Moreover, BATF, activated by LPS/IL-4/IL-6, plays a significant role in B cell differentiation, antibody production, the generation of regulatory B cells, and the regulation of inflammation [6, 16]. While research on BATF2 and BATF3 is limited, it is known that BATF2 is expressed in M1 macrophages and Th1 cells and can enhance the expression of pro-inflammatory factors in macrophages [36, 37], aiding in the early defense against Klebsiella pneumoniae infections [38, 39]. BATF3 is primarily expressed in CD8^+^dendritic cells and is essential for the development of resident lymphoid organ CD8α^+^dendritic cells and migratory CD103^+^dendritic cells found in the intestines, lungs, and dermis, participating in cytokine-mediated T cell responses against viruses and tumors [40, 41].

### Binding and modification of BATF

BATF, as a member of the bZIP family, can form heterodimers with AP-1 and its members (such as c-Jun and Fos) to jointly regulate the expression of multiple immune-related genes [18]. The BATF-AP-1 complex plays a central role in the regulation of immune cell function, while its cooperation with the IRF family further expands its role in immune cell differentiation and function [3]. The IRF family is another important group of transcription factors that primarily regulate immune responses, antiviral responses, and inflammatory reactions [42, 43]. The mammalian IRF family includes nine members, from IRF1 to IRF9, among which IRF4 and IRF8 can form complexes with BATF family transcription factors [15]. IRF4 is expressed exclusively in the immune system and must form heterodimers with other factors to strongly bind to the DNA sequence GAAA [44]. These interactions occur through direct interactions, co-binding with regulatory regions of specific genes, or influencing each other’s transcriptional activity [3, 45]. Structurally, the C-terminal of the IRF-associated domain (IAD) in IRF4 and IRF8 interacts with the leucine zipper domain of BATF, activating its biological functions [15]. The BATF/Jun heterodimer can form a trimeric complex with IRF4 or IRF8, which binds to a shared sequence called AICE [45]. The trimeric complexes formed by BATF with AP-1 and members of the IRF family not only play a role in the activation of immune responses but also have important functions in processes such as cell fate determination, immune tolerance, and antitumor immunity [4, 30, 46–48].

The phosphorylation modifications of BATF also play an important role in regulating its function, in addition to its interactions with the AP-1 and IRF families. The impact of BATF on DNA binding and gene regulation through serine-43 phosphorylation. BATF can form heterodimers with Jun proteins and localize to the nucleus; however, studies have found that Ser43 phosphorylation prevents DNA binding. Nevertheless, BATF’s ability to inhibit AP-1-mediated gene transcription remains unaffected, indicating that this function is independent of DNA binding. Additionally, phosphorylated BATF(S43D) heterodimers do not show significant effects on nuclear localization or dimerization with Jun. The study further reveals that 40% of bZIP proteins contain potential phosphorylation sites similar to serine-43, suggesting a similar regulatory mechanism. Compared to the commonly observed redox regulation of bZIP proteins, this study highlights phosphorylation as another crucial regulatory mechanism for bZIP factors. Future research should identify the kinase responsible for BATF phosphorylation and its regulatory conditions, as well as explore the role of this modification in other bZIP factors and its impact on gene expression. This provides a new perspective on the signaling regulation and functional mechanisms of bZIP proteins [49–51].

### BATF family compensation mechanism

The BATF family members (including BATF, BATF2, and BATF3) play crucial roles in regulating immune system functions, particularly in the differentiation, activation, and immune responses of immune cells. Although these three proteins share some structural and functional redundancy, their roles differ across various cell types. The compensatory mechanisms between BATF family members can partially or fully restore immune function in the case of deficiencies or dysfunctions [52].

The functions of BATF, BATF2, and BATF3 in DCs are closely related, and the compensatory mechanisms between them ensure that the immune system maintains functional stability even in the absence of one of the members. BATF3 plays a crucial role in the development of CD103^+^and CD8α^+^dendritic cells [53, 54]. Mice deficient in BATF3 exhibit significant defects in dendritic cell function, leading to a weakened T cell immune response [53, 55]. However, BATF can partially compensate for the loss of BATF3, restoring dendritic cell function, especially during infections [3, 52]. For example, results from Roxane Tussiwand and colleagues show that both in vitro and in vivo experiments demonstrate that BATF3-deficient cells or mice retain a portion of the normal CD8α^+^DC population and function, allowing them to resist infections. Mice deficient in both BATF and BATF3 lack CD8α^+^DC, are unable to present antigens, and fail to initiate cellular immune responses to kill viruses [52].

BATF2 and BATF3 have some overlap in the differentiation of immune cells, particularly in the immune response of dendritic cells. In the case of Toxoplasma gondii infection or IL-12 induction, BATF2-deficient mice show a reduction in the CD103^+^DC population in the lungs and exhibit a weakened immune tolerance in dendritic cells [52]. Similar to BATF, BATF2 can partially compensate for the CD103^+^DC defects caused by BATF3 deficiency, helping to maintain immune tolerance [52]. Further research on this compensatory mechanism suggests that the cross-compensation between BATF family members primarily occurs through the leucine zipper (LZ) domain, rather than the DBD, by interacting with non-AP-1 family members [52].

BATF3 can restore certain functions in the absence of BATF, such as IL-17 production by Th17 cells, CTLA-3 and IL-10 production by Th2 cells [52, 56]. In mucosal inflammation, the differentiation defect of Th9 cells and the deficiency in IL-9 production in BATF knockout mice can be compensated by TL1A. TL1A induces BATF3 expression and promotes its binding to the IL-9 promoter, thereby increasing IL-9 secretion [29]. In mouse heart transplantation experiments, it was found that mice lacking both BATF and BATF3 (Batf^−/−^Batf3^−/−^mice) spontaneously accepted long-term heart allografts. However, the individual deficiency of BATF or BATF3 alone did not prevent graft rejection [57].

During B cell class switch recombination (CSR), BATF and BATF3 also have overlapping functions. When BATF is deleted, the presence of BATF3 partially restores B cell function, ensuring the integrity of the immune response [52].

Understanding the compensatory mechanisms between BATF family members not only helps to reveal the regulatory mechanisms of immune cell function but is also crucial for maintaining immune tolerance and regulating the onset of inflammatory responses.

## BATF regulates immune cells differentiation and function

### Innate immune cells

#### Innate lymphoid cells (ILCs)

ILCs are classified into ILC1, ILC2, and ILC3 based on the transcription factors they express—T-bet, GATA3, and RORγT—mirroring the categorization of Th1, Th2, and Th17 cells [58]. These cells are rare in peripheral blood but predominantly reside in tissues such as the skin, respiratory tract, and gastrointestinal tract [58]. Research indicates that BATF plays a crucial regulatory role in the development and differentiation of ILCs. IL-7 signaling induces the expression of BATF in ILC progenitors through the STAT3/6 pathway [25]. BATF expression is particularly prominent in bone marrow ILC progenitors and mature ILC subsets in the small intestine, especially highest in αLP and iLCP (ILC precursors) [25]. Compared to CD4/CD8 T cells, γδ T cells, B cells, dendritic cells, and macrophages, BATF expression is more pronounced in various ILC subsets. BATF supports the hematopoiesis of ILCs by promoting the transition from CLPs to ILC progenitors and is vital for the normal development of most ILC groups, including ILC1, ILC2, and ILC3. Furthermore, BATF is essential for the proliferation of peripheral ILCs and the production of effector cytokines. In the absence of BATF, there is a significant reduction in the number of ILC progenitors at the αLP stage. The efficiency of αLP, CHILP, iLCP, ILC2P, and ILC1P cells in the bone marrow decreases, and the production of mature ILC1, ILC2, and ILC3 cells is affected in the spleen, liver, lungs, mesenteric lymph nodes (MLN), and small intestine (SI). Mechanistic studies have shown that the expression of the NFIL3 transcription factor significantly decreases in αLP cells with a BATF deficiency. Supplementing NFIL3 significantly enhances the differentiation of ILC cells, indicating that BATF mediates the differentiation of early ILC cells through the regulation of NFIL3 [25].

ILC1 cells primarily produce interferon-γ (IFN-γ) in response to IL-12, which is crucial for combating intracellular microbial infections [59]. In BATF-deficient mice, while IFN-γ production by ILC1 cells in the spleen and colon remains unaffected, there is a significant reduction in IFN-γ expression in ILC1 and NK cells in the liver and small intestine. This finding suggests that BATF is essential for regulating the activity and function of ILC1 cells, thereby influencing the body’s immune response to microbial invasions. ILC2 cells are activated by IL-33, IL-25, and thymic stromal lymphopoietin (TSLP), leading to the production of cytokines such as IL-4, IL-5, and IL-13, which are involved in allergic responses and defense against parasitic infections [60]. During influenza virus infection, BATF deficiency impairs ILC2 cell differentiation, weakening immune protection and exacerbating disease severity [61]. Additionally, BATF deficiency results in reduced cytokine production by ILC2 cells. In mouse models, ILC2 cells are categorized into tissue-resident natural ILC2 (nILC2) and migratory inflammatory ILC2 (iILC2). IL-25 specifically promotes the expansion of the iILC2 subset, enhancing resistance to worm infections. In worm infection models treated with IL-25, BATF deficiency impairs iILC2 cell production [62]. Migratory iILC2 cells, which are early sources of IL-4 and IL-13 in the lungs and intestines, are distinct from tissue-resident nILC2 cells. Migratory iILC2 cells express low levels of the ILC2 marker arginase 1 (Arg1) and play a key role in early responses to damaged mucosal epithelia, facilitating barrier reconstruction at affected sites. In type 2 respiratory diseases, CD45RA^+^ILC2 cells, stimulated by IL-33 and TSLP, upregulate BATF and IRF4, activating the expression of hnRNPLL and leading to the formation of CD45RO^+^ILC2, which resembles iILC2 cells in mice. Clinically, corticosteroid medications are commonly used to treat type 2 respiratory diseases mediated by these cells [63]. Therefore, BATF plays a critical role in ILC2 cells by modulating cell development, function, and immune regulation. ILC3 cells are induced by IL-7, IL-23, and IL-1β and produce cytokines such as IL-22, IL-17 A, GM-CSF, and IFN-γ, which help combat fungi and extracellular microbes [64]. In ILC3 cells, BATF collaborates with c-Maf in NCR (natural cytotoxicity triggering receptor) positive ILC3 to regulate RORγT expression, thus promoting the ILC3 phenotype. In Clostridium difficile-infected mouse models, BATF deficiency reduces the number of RORγT-positive ILC3 cells in the small intestine and decreases IL-22 production. Additionally, BATF influences ILC3 cell development at various sites, such as by inducing MHC-II expression on small intestine ILC3 cells to suppress CD4^+^T cell activation [65].

In summary, BATF regulates the development and differentiation of ILC3 cells through interactions with various transcription factors, thereby influencing their phenotypic and functional characteristics. This regulatory mechanism is essential for both the maturation and differentiation of ILC3 cells and their subsequent role in immune regulation (Table 1).

#### Dendritic cells

Dendritic cells (DCs) are hematopoietic cells that play a crucial role in linking innate and adaptive immune responses [66]. DC progenitors migrate through the blood to various tissues and lymphoid organs, where they differentiate into distinct subtypes, including CD8α^+^DCs, CD103^+^DCs, and CD11b^+^DCs [66]. Among these subtypes, CD8α^+^DCs are essential for mediating tolerance to self-antigens in peripheral tissues through direct interactions with autoreactive T cells [66]. Research has found that BATF is highly expressed in activated pDCs [67]. During activation, the transcription factor BATF remodels the chromatin landscape and reprograms gene transcription in pDCs. Additionally, by directly binding to the Zfp366 gene region, it induces the expression of the transcription regulator DC-SCRIPT, thereby inhibiting the transcription of IFN I genes in pDCs [68]. BATF3 is a pivotal factor in the development of CD8α^+^DCs; its absence leads to impaired CD8α^+^DC formation, primarily due to intrinsic hematopoietic defects [53]. Research indicates that BATF3 deficiency affects the cross-presentation capability of CD8α^+^DCs during infections, resulting in functional impairment [53]. In BATF3-deficient mice, bone marrow-derived DCs fail to produce IL-12 upon Toll-like receptor 3 (TLR3) stimulation, and these mice exhibit significantly weakened CD8^+^T cell responses to West Nile virus and tumor-specific CD8^+^T cell responses to syngeneic fibrosarcoma [53]. Furthermore, in mouse models infected with Leishmania, BATF3 deficiency severely impairs the formation of both CD8α^+^DC and CD103^+^DC subtypes [69]. These mice display more severe skin lesions, higher parasite loads, and reduced IFN-γ production in their draining lymph nodes, leading to diminished disease resistance [69, 70]. These findings highlight the critical role of BATF3 in the development and antigen-presentation functions of CD8α^+^DCs and CD103^+^DCs (Table 1).

### Lymphocytes

#### Th17 cells

The differentiation of Th17 cells is initiated by the stimulation of IL-6, IL-21, and TGF-β, which leads to the upregulation of RORγT and RORα and subsequent production of cytokines including IL-17 A, IL-17 F, and IL-22 [71, 72]. Th17 cells are pivotal in the pathogenesis of various inflammatory and autoimmune diseases, such as MS, RA, psoriasis, and IBD [73, 74].

**Fig. 2:**
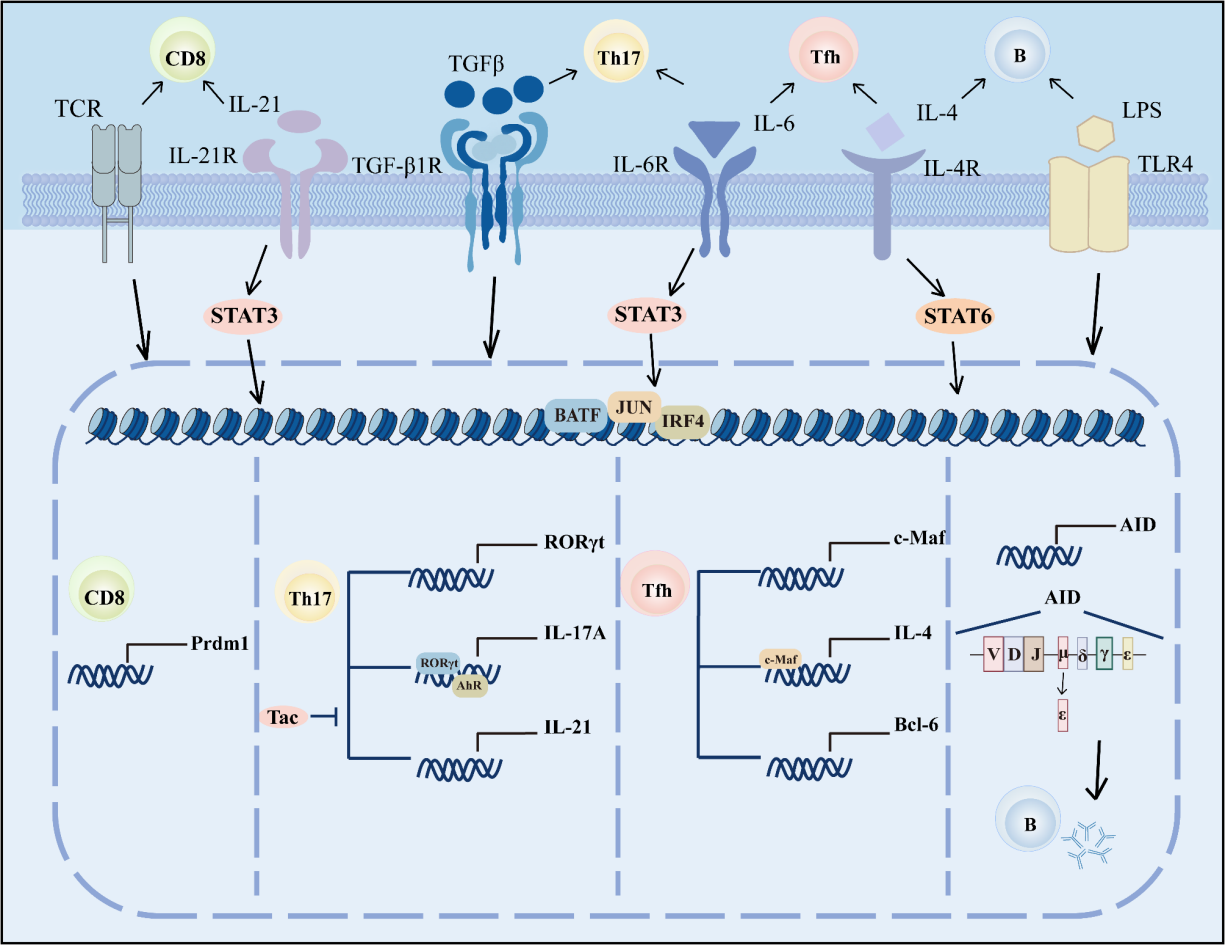
BATF is crucial for lymphocyte differentiation. IL-6 and TGF-β promote the formation of the BATF/Jun/IRF4 trimeric complex (hereafter referred to as the AP-1 complex), facilitating Th17 cell differentiation. This complex can activate the expression of cytokines and transcription factors such as RORγt and IL-21. Tac can reduce IL-21 production by Th17 cells by inhibiting the formation of the AP-1 complex. Additionally, RORγt and AhR binding to the trimer further promote the expression of IL-17 A. Similarly, IL-6 and IL-4 can induce the formation of the AP-1 complex, promoting Tfh cell differentiation and activating transcription factors such as c-Maf and Bcl-6. C-Maf can also bind to the AP-1 complex to promote the production of IL-4 cytokines. TCR and IL-21 promote Prdm1 transcription via the AP-1 trimer, facilitating early memory formation in CD8^+^T cells. Furthermore, IL-4 and LPS can promote the formation of the AP-1 complex, thereby enhancing AID gene expression, leading to B cell maturation and antibody production

Research indicates that under Th17 polarizing conditions, BATF mediates the differentiation of Th17 cells by directly binding to the promoter and intergenic regions of genes such as IL-21 and recruiting CTCF [75, 76]. Studies by Barbara U. Schraml and colleagues have demonstrated that BATF-deficient mice exhibit significant impairments in Th17 cell differentiation, with key factors required for Th17 differentiation, such as RORγT and IL-21, being undetectable [4]. Even with the addition of IL-21 or overexpression of RORγT in vitro, IL-17 expression could not be restored in these mice [4]. These findings underscore the essential role of BATF in Th17 cell differentiation and its direct regulation of IL-17 expression. Additionally, BATF-deficient mice show reduced expression of the IL-23 receptor (IL-23R) in Th17 cells, which is crucial for their proliferation and survival [4]. Despite normal TGF-β signaling and unchanged STAT3 phosphorylation levels induced by IL-6, these observations suggest that BATF may influence Th17 cell differentiation by affecting downstream target genes within the IL-6 signaling pathway [4, 77] (Fig. 2).

During Th17 cell differentiation, BATF primarily forms heterodimers with JunB, binding to the promoter and intergenic regions of IL-17 A to exert its effects [78]. Research by Maria Ciofani and colleagues has shown that T-cell receptor (TCR) stimulation significantly enhances the binding of BATF to IRF4 in cis-regulatory regions. This interaction also strengthens the binding of BATF with other key transcription factors involved in Th17 cell differentiation, including c-Maf, STAT3, RORγT, and p300, highlighting the crucial role of the BATF-IRF4 interaction in Th17 cell differentiation [14]. Furthermore, the Ets1-Runx1 complex has been identified as a promoter of Th1 and Treg cell development. BATF counteracts the formation of this complex by inhibiting IL-2 signaling and its subsequent activation of STAT5, thus stabilizing the Th17 cell differentiation state [79]. In studies involving worm infections, IRAK-2 (interleukin-1 receptor-associated kinase-like 2) has been shown to enhance Th17 cell development by augmenting IL-1-induced activation of RORγT and BATF, which may contribute to disease exacerbation [80]. From a metabolic standpoint, mitochondrial respiration is increased during Th17 cell development, with mitochondrial oxidative phosphorylation promoting BATF expression [81]. Notably, specific inhibition of mitochondrial ATP synthase impairs Th17 cell development and function, although this effect can be counteracted by BATF overexpression [81] (Table 1).

These studies indicate that BATF plays an indispensable role in maintaining the differentiation and function of Th17 cells.

#### Tfh cells

The differentiation of Tfh cells is predominantly regulated by IL-21-induced activation of STAT3, with Bcl-6 serving as the principal transcription factor [82]. In autoimmune diseases such as SLE, RA, and primary Sjögren’s syndrome (pSS), Tfh cells contribute to the dysregulated proliferation of B cells and the production of autoantibodies [83].

Research indicates that BATF is highly expressed in Tfh cells and directly binds to the promoter regions of Bcl-6 and other Tfh-associated genes, such as c-Maf and IL-21 [16]. The absence of BATF impairs Tfh cell differentiation. Studies by Anupama Sahoo and colleagues have demonstrated that BATF mediates IL-4 expression in Tfh cells [5]. BATF forms a complex with IRF4 and interacts with STAT3 and STAT6 to bind to the non-coding sequence 2 (CNS2) region of the IL-4 gene, thereby promoting IL-4 production [5]. Additionally, BATF influences the abundance of chromatin markers, such as AcH3 and H3K4, in the CNS2 region of Tfh cells [5]. The IL-6/STAT3 and IL-4/STAT6 signaling pathways are also involved in regulating BATF expression in Tfh cells, thereby enhancing IL-4 production [84]. During the CD4^+^CD8^+^double-positive stage, Bcl-6 is transiently upregulated and binds to the E3 ligase cullin3, which inhibits the expression of BATF and Bcl-6 [85]. This Bcl-6-CULLIN3-mediated negative feedback mechanism modulates the Tfh response [85]. Upon migration to B cell follicles, Tfh cells experience transient and sustained calcium signaling triggered by the binding of ICOS to ICOSL. Store-operated calcium entry (SOCE), mediated by intracellular calcium stores, plays a dual role in both antibody-mediated protective immunity and autoimmunity [86]. SOCE regulates the differentiation of T follicular regulatory (Tfr) and Tfh cells by modulating the expression of transcription factors NFAT, IRF4, BATF, and Bcl-6, thereby inhibiting the production of autoantibodies [86].

In an allergic asthma model, BATF-deficient mice exhibited reduced symptoms of asthma, including a decrease in eosinophils and lymphocytes, and a significant reduction in cytokines such as IL-4, IL-5, and IL-13 in the lungs [5]. These findings suggest that BATF is critical for regulating IL-4 production in Tfh cells, thereby influencing the development of allergic asthma. Regarding liver transplantation, Tfh cells are implicated in transplant rejection by promoting B cell activation and antibody production through cytokines like IL-21 [87]. Clinically, tacrolimus treatment reduces the formation of the BATF/Jun/IRF4 complex, which leads to decreased IL-21 production, a lower proportion of Tfh cells, and reduced expression of Bcl-6 and IL-6 [87]. This effect contributes to improved survival rates of transplanted livers and mitigates transplant rejection [87] (Fig. 2; Table 1).

In summary, BATF influences the entire process of humoral immune response by regulating the differentiation, function, and interactions of Tfh cells with other immune cells.

#### Th2 cells

Th2 cell differentiation is driven by IL-4/STAT6 and IL-2/STAT5 signaling pathways and results in the production of cytokines such as IL-5, IL-9, IL-13, and IL-24, which are critical for immune responses to extracellular parasites and in the context of allergic diseases [82]. BATF is pivotal in modulating the production of these type II cytokines, particularly IL-4, within Th2 cells. In murine models of worm infection, BATF deficiency leads to impaired Tfh and Th2 cell production, as well as diminished cytokine production, including IL-4 and IL-13, by CD4^+^T cells [88]. Research has demonstrated that BATF is prominently associated with AP-1 site regulatory regions of type II cytokine gene loci in Th2 cells, notably at Rad50 hypersensitive sites (RHS) 6 and RHS7 [88]. Furthermore, BACH2 plays a role in modulating immune responses by preventing excessive activity that could lead to autoimmune diseases. The Bach2-BATF complex exhibits functional antagonism with the IRF4-BATF complex, influencing Th2 cell differentiation and cytokine production [89]. This antagonism is notably diminished in BATF-deficient CD4^+^T cells, underscoring the critical role of BATF in Th2 cell differentiation and function. Additionally, BACH2 reduces the expression of BATF and BATF3 through regulation of IL-4 [90] (Table 1).

Overall, BATF influences the differentiation and function of Th2 cells by regulating gene transcription activity. It is involved in controlling the expression of Th2 cell surface markers, cytokine secretion (such as IL-4, IL-5, IL-13, etc.), and intercellular interactions, thereby determining the functional performance of Th2 cells.

#### Th9 cells

Th9 cells differentiate from naive T cells in response to the combined stimulation of TGF-β and IL-4, with their primary function being the production of IL-9, a cytokine associated with Th2 cell responses [82]. Studies have demonstrated that BATF is essential for the function of Th9 cells, particularly in the context of allergic asthma [28]. Moreover, Th9 cells play significant roles in the pathogenesis of various diseases, including EAE, hypersensitivity pneumonitis, and IBD [29].

BATF forms a complex with IRF4 to activate the expression of the IL-9 gene. The absence of BATF significantly reduces the expression of Th9 cell-related genes, as evidenced by mouse models demonstrating decreased incidence of experimental asthma [91]. Conversely, augmenting BATF expression can partially restore IL-9 levels [91]. Additionally, IRF8 collaborates with BATF to further enhance IL-9 transcription [92]. Studies indicate that TL1A, a member of the tumor necrosis factor superfamily, induces Th9 cell differentiation and increases the expression of BATF and BATF3 in a STAT6-dependent manner. These factors bind to the IL-9 promoter region, with BATF3 specifically promoting IL-9 production in Th9 cells [29, 93]. Furthermore, 1,25-dihydroxyvitamin D3 reduces BATF expression by inhibiting AhR, consequently decreasing IL-9 production in Th9 cells [94]. Research also shows that Toll-like receptor 2 (TLR2) promotes Th9 cell differentiation by upregulating BATF transcription [95]. In the elderly, naive CD4^+^T cells exhibit increased expression of BATF and IRF4, while the expression of ID3 and Bcl-6 decreases, facilitating Th9 cell differentiation [96] (Table 1).

In summary, BATF plays a regulatory role in Th9 cells by modulating the production of IL-9 and the expression of other cytokines, impacting the processes of immune response, inflammatory response, and immune balance.

#### Treg cells

Regulatory T (Treg) cells primarily maintain autoimmune tolerance and are involved in the pathogenesis of autoimmune diseases, tumors, and other conditions [97]. Effector Treg (eTreg) cells are found in non-lymphoid tissues and tumors [98]. Research indicates that BATF plays a crucial role in the differentiation and maintenance of eTreg cells, with BATF deficiency affecting their generation and proliferation [99]. In melanoma patients, T cells express a large number of Treg immunosuppressive and stability-maintaining molecules under the influence of transcription factors such as FOXP3 and BATF, promoting Treg cell polarization. Additionally, new Treg functional molecules (SPOCK2, SH2D2A) and ligand molecules (ITGB2, LTA, CLEC2C, CLEC2D) were identified, which interact with FOXP3 or BATF [100]. BATF is necessary for the migration of Treg cells to non-lymphoid tissues. The non-lymphoid Treg precursors refer to T cell precursor populations found in non-lymphoid tissues that have regulatory potential [101]. BATF and IRF4 directly or indirectly regulate the differentiation and immunosuppressive function of Treg cells, as well as the differentiation and development of tissue-resident Treg cells, promoting the generation of non-lymphoid Treg precursors [33, 102]. Studies have shown that Treg cells in the lung cancer tumor microenvironment (TME) and those in peripheral blood exhibit different chromatin landscapes, and sequencing has identified BATF as a key differentiation-related transcription factor for Treg cells in the TME [103]. Tissue-resident and tumor-invasive Tregs share common differentiation and activation pathways, with most differentiation and activation of Tregs in tissues and tumors being mediated by BATF [103]. High expression of BATF in Treg cells is associated with poor cancer prognosis [103]. Conversely, BATF3 is considered a transcriptional inhibitor of Treg cell differentiation [104]. BATF3 and IRF4 bind to the FOXP3 locus to inhibit FOXP3 expression and the production of FOXP3^+^Tregs (iTregs) [105]. Studies have shown that OX40 (CD134) upregulates BATF3 to inhibit Foxp3 expression in a Sirt1/7-dependent manner [106] (Table 1).

In summary, BATF regulates the differentiation, function, and tissue residency of Treg cells through multiple mechanisms, thereby playing a crucial role in maintaining immune tolerance and preventing excessive immune responses.

#### BATF affects CD8+T cell differentiation

BATF not only functions in CD4^+^T cells but also plays a crucial role in the functionality of CD8^+^T cells [7]. Researchers such as Makoto Kurachi have discovered that BATF is essential for the early differentiation of effector CD8^+^T cells [7]. Their studies show that within 24 h after activation by both primary and secondary signals, BATF binds to genes involved in the differentiation of effector CD8^+^T cells [7]. In primary CD8^+^T cells lacking BATF, these cells are unable to differentiate normally into effector CD8^+^T cells [107]. For instance, following infection with Lymphocytic Choriomeningitis Virus (LCMV), the expression levels of BATF significantly increase in the infected effector CD8^+^T cells and persist in memory CD8^+^T cells [7]. Additionally, studies indicate that P14 cells (a type of CD8^+^T cell specific to the LCMV gp33 epitope presented on H-2Db) lacking BATF exhibit defects in differentiation into effector CD8^+^T cells in response to infection with monocytotropic Listeria monocytogenes or dendritic cell immunization stimulated by peptides [7]. These findings underscore the necessity of BATF for the functionality of effector CD8^+^T cells.

BATF functions in effector CD8^+^T cells by forming heterodimers with molecules such as c-Jun, JunB, and JunD [7]. These heterodimers primarily bind with IRF4 and are concentrated in the AICE sequence region [7, 108] (Fig. 2). Research has shown that BATF can bind to and promote the expression of various transcription factors related to effector differentiation, such as T-bet, Blimp-1, and Runx3, as well as cytokine receptors and signaling molecules like IL-2R and STATs [7]. Concurrently, BATF can also inhibit downstream molecules of these transcription factors and cytokine signaling pathways, such as IFN-γ and granzyme B [7]. This indicates that BATF has a dual role in both upregulating effector transcription factors and suppressing the expression of effector molecules, a phenomenon known in biology as a ‘coherent feedforward loop,’ which helps to inhibit untimely responses, thereby stabilizing the differentiation of effector cells [7]. In patients infected with HIV, it has been found that progressors exhibit higher BATF expression in exhausted HIV-specific CD8^+^T cells compared to controllers, while no significant difference is observed in their naive CD8^+^T cells [109]. This phenomenon may suggest that BATF can regulate the function of effector CD8^+^T cells depending on the intensity and duration of TCR stimulation.

The deletion of BATF leads to failed differentiation of effector CD8^+^T cells, which may be associated with epigenetic reprogramming and energy metabolism [110]. For instance, Shoko Kuroda and colleagues found that IL-12-induced BATF mediates the differentiation of effector CD8^+^T cells through histone acetylation and chromatin remodeling. Additionally, BATF promotes histone acetylation at the T-bet locus and ATP production by inhibiting the expression of the NAD^+^-dependent deacetylase Sirt1, thereby driving the differentiation and survival of effector cells [110]. In summary, BATF facilitates the differentiation of effector CD8^+^T cells by regulating Sirt1 expression, epigenetic restructuring, and energy metabolism, highlighting its critical role in the metabolic processes of cell differentiation. In the context of chronic infections, the functionality of CD8^+^T cells depends on IL-21 secreted by CD4^+^T cells [111]. Researchers such as Battegay (1994) discovered that in the absence of CD4^+^T cell help, CD8^+^T cells gradually become exhausted and lose their functionality [112]. Subsequent studies, including the work by Elsaesser (2009), confirmed that IL-21 produced by CD4^+^T cells is crucial for maintaining the antiviral activity of CD8^+^T cells [113]. More specifically, Gang Xin (2015) found that IL-21 promotes the sustained expression of BATF in CD8^+^T cells, which, when combined with IRF4, maintains the expression of Blimp-1, thereby enabling CD8^+^T cells to continue functioning [111]. Additionally, IL-21 can induce the expression of the functional receptor IL-1RI, enhancing the effector function of CD8^+^T cells through the regulation of BATF, IRF4, and Blimp-1 [114].

In chronic infections, CD8^+^T cells are classified into three subgroups: Ly108^+^Tcf-1^+^progenitor cells, Ly108^−^CX3CR1^−^terminally exhausted cells, and CX3CR1^+^cytotoxic effector cells [115]. Research by Chen and colleagues indicates that CD4^+^T cells contribute to the differentiation of progenitor cells into CX3CR1^+^cytotoxic effector cells rather than terminally exhausted CD8^+^T cells, a process involving the binding of BATF with IRF4 [115]. BATF also plays a significant role in transplant rejection and cancer treatment contexts [48, 116]. CD8^+^T cells lacking BATF fail to effectively infiltrate transplants and do not produce the cytokines and effector molecules necessary to attack the graft [116]. Conversely, CAR T cells expressing BATF combined with IRF4 exhibit enhanced anti-tumor functions and proliferative capabilities [117].

However, studies have also shown that BATF and IRF4 expression are similarly elevated in exhausted CD8^+^T cells [118]. Specifically, BATF regulates the expression of immune checkpoint genes (such as PD-1 and LAG-3) by cooperating with members of the AP-1 family, while IRF4 further promotes the exhausted state of CD8^+^T cells by enhancing the transcription of these genes [118]. Michael Quigley and his colleagues found that during HIV infection, antigen-specific CD8^+^T cells suppress T cell function through PD-1-mediated upregulation of BATF, and knocking out BATF can restore the function of HIV-specific T cells [109]. In the context of tuberculosis, BATF mediates the negative regulation of T cell function through the PD-1/PD-Ls pathway [119]. In cancer models, BET(bromodomain and extra-terminal motif) inhibitors have been shown to positively impact CD8^+^T cells by directly inhibiting BATF, thereby enhancing the persistence of stem-like memory CD8^+^T cells [120].

The combined binding of IRF4, BATF, and NFAT collectively regulates genes encoding components involved in cellular metabolism and the mitochondrial electron transport chain, thereby contributing to the regulation of CD8^+^T cell exhaustion [118]. Studies have shown that in terminally exhausted cells, IRF: Batf motifs are highly enriched and significantly overlap with Tox [121].

For another member of the BATF family, BATF3, it increases the accessibility of memory-related genes (such as TCF7, MYB, IL7R, CCR7, and SELL) and effector-related genes (such as EOMES and TBX21), while reducing the accessibility of exhaustion-related gene loci (such as TIGIT, CTLA4, and LAG3), thereby counteracting CD8^+^T cell exhaustion [122].

In summary, the role of BATF in CD8^+^T cell immune responses is dual in nature, as it performs distinct functions at different stages of the immune response. During the formation of early effector CD8^+^T cells and the later promotion of CD8^+^T cell exhaustion, BATF exhibits a complex regulatory pattern. BATF not only plays a critical role in the initial expansion and establishment of effector functions in CD8^+^T cells but also drives immune tolerance and T cell exhaustion during chronic immune responses. This duality indicates that BATF plays an important yet delicate role in T cell immune regulation. Understanding the specific mechanisms of BATF in CD8^+^T cell exhaustion provides new opportunities to improve clinical immunotherapies, such as immune checkpoint inhibitors and cancer immunotherapy (Table 1).

#### BATF regulates B cell function

B cells play a critical role in autoimmune diseases such as SLE and pSS, where their function is regulated by cytokines secreted by Tfh cells [123]. These cytokines stimulate B cell proliferation and the production of antibodies that target self-antigens [124–126]. The transcription factor BATF is particularly crucial in B cells. In mouse models deficient in BATF, although B cell proliferation remains normal, these mice fail to form germinal centers after antigen stimulation, and antibody class switching is impeded [46]. While the levels of μ germline transcripts (GLT) in these mice are similar to those in normal mice, levels of IgM are slightly reduced, and other types of GLT are significantly decreased, with other immunoglobulin types almost undetectable. In vitro experiments also did not detect the Aicda gene, which encodes activation-induced cytidine deaminase (AID) [6] (Fig. 2). These findings suggest that BATF is involved in the formation of germinal centers and antibody class switching in B cells. Blocking BATF within 12 h of B cell stimulation results in the absence of Aicda gene detection after 72 h, and no detection of IgG1 and IgE antibodies after 96 h [46]. However, if BATF is blocked 12 h after B cell stimulation, these indicators remain unchanged, indicating that early expression of BATF is necessary for antibody class switching [46]. BATF with IRF4 binds to AICE motifs. IRF4 can bind to Ets composite motifs (EICE) together with the PU.1 or SpiB Ets factors. This complex cooperatively regulates genes associated with B cell activation and the germinal center response [127]. BATF also promotes the recruitment of 5-hydroxymethylcytosine (5hmC) mediated by Tet enzymes to the Aicda enhancer, inducing AID expression and thus promoting CSR [128] (Table 1).

In summary, B cells expressing BATF can normally undergo antibody class switching and form germinal centers.

## The role of BATF in autoimmune diseases

### Inflammatory bowel disease

**Fig. 3:**
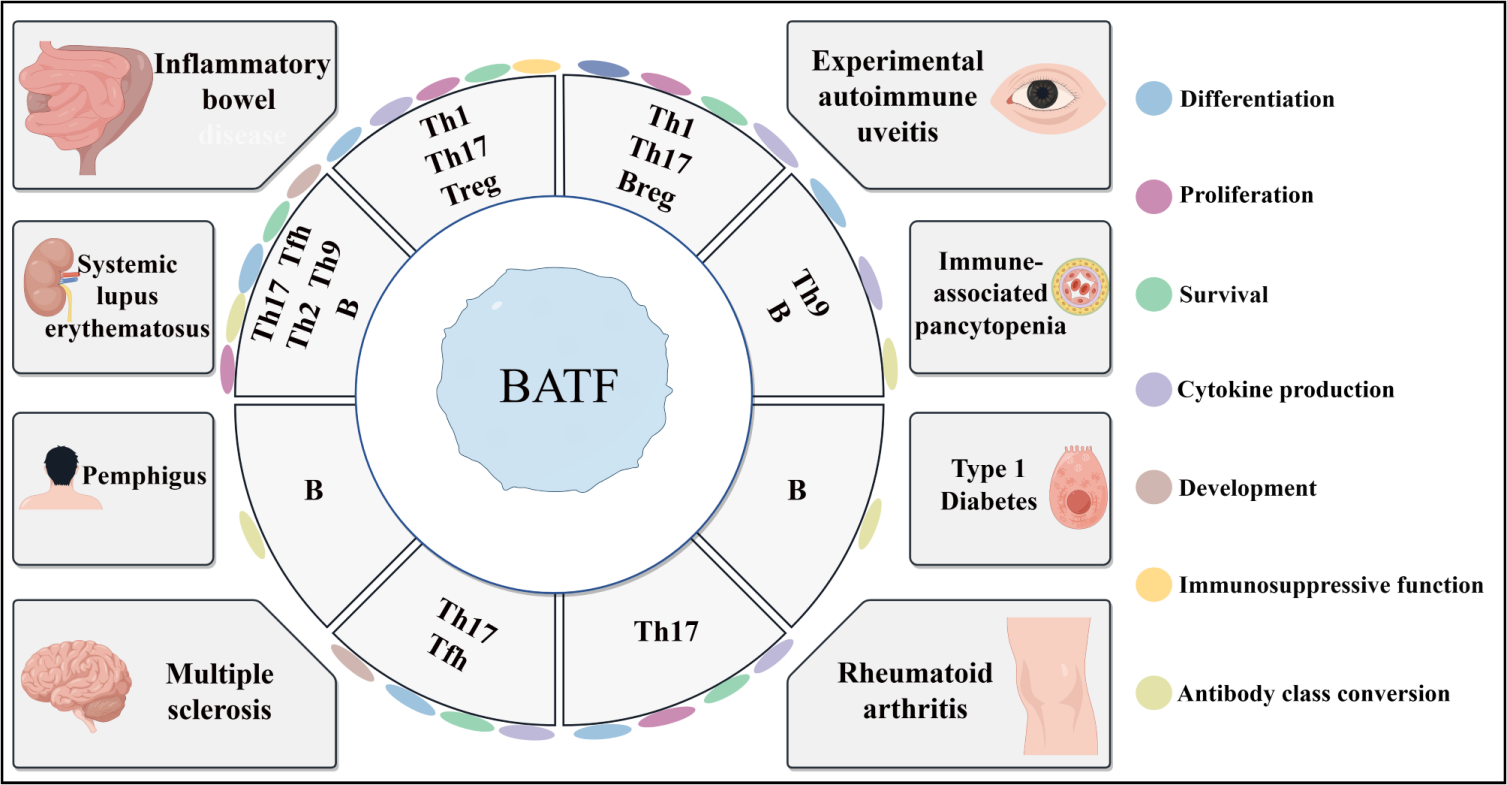
BATF plays a significant role in autoimmune diseases. In a mouse model of MS, BATF is closely associated with the differentiation of Th17 and Tfh cells. Mice lacking BATF show milder disease symptoms, suggesting that BATF promotes MS progression. In patients with SLE, BATF expression is upregulated in T cells and B cells. By regulating the activation and function of these cells, BATF may contribute to the development of SLE. In RA studies, BATF may influence RA progression by regulating the differentiation and function of Th17 cells. In IBD, BATF significantly affects the roles of Th17 and Treg cells. BATF may impact the course of IBD by influencing their differentiation and function. Additionally, BATF promotes Th17 cells in experimental autoimmune uveitis, autoreactive B cells in pemphigus and T1D, and Th9 cells in IRP, thereby exacerbating the progression of autoimmune diseases. By Figdraw

IBD, encompassing Crohn’s Disease (CD) and Ulcerative Colitis (UC), consists of chronic, recurrent intestinal conditions typically marked by rectal bleeding and weight loss [129]. Historically, CD has been associated with Th1 cells producing IFN-γ, while UC has been linked to Th2 cells producing IL-4 and IL-13 [129]. However, recent research suggests that Th17 cells also play a role in IBD progression, particularly through targeting RORγT, as evidenced by the effective suppression of colitis in mouse models [10]. BATF, a critical regulator of Th17 cell differentiation, is significantly upregulated in IBD [10]. Notably, in UC patients, increased disease severity correlates with heightened BATF expression in inflamed colon tissues, alongside elevated levels of IL-23 p19 and IL-23R [130]. Studies using a dextran sulfate sodium-induced colitis mouse model have identified numerous IL-17^+^neutrophils that promote disease progression via an IL-23 and STAT3-dependent RORγT and BATF pathway [131]. In a T-cell transfer model of colitis, Th17 cells deficient in BATF failed to induce IBD [10]. Additionally, in a colitis-associated cancer (CAC) model, BATF-deficient mice exhibited reduced tumor numbers and sizes, indicating that BATF-dependent IL-23R^+^IL-6^+^CD4^+^Th17 cells are pivotal in colitis-associated tumorigenesis [130]. In UC patients, FOXP3^+^T cells expressing BATF have been observed [132]. In mouse models, BATF regulates the tissue residency of Treg cells by controlling CCR9 and α4β7 expression in the intestine. BATF-deficient mice show reduced intestinal FOXP3^+^T cells and their effector molecules, underscoring BATF’s role in promoting FOXP3^+^T cell differentiation in the intestine and inhibiting disease progression [133]. Additionally, IBD patients exhibit high numbers of ILCs, particularly ILC3 cells, which promote IBD progression [134]. The nuclear circRNA CircKcnt2 inhibits BATF expression by recruiting the NuRD complex to the BATF promoter, thereby suppressing IL-17 expression and reducing ILC3 activity, aiding in the resolution of innate colitis [12]. Furthermore, in vitro colonic organoid cultures lacking BATF displayed normal development, morphology, and function, indicating that intestinal crypts are not regulated by BATF [10] (Fig. 3; Table 2).

In conclusion, targeting BATF not only results in the loss of pathogenic T cell activity but also preserves the development and differentiation of intestinal epithelial cells, presenting a promising approach for treating IBD.

### Multiple sclerosis

MS is an autoimmune disease characterized by inflammatory demyelination in the white matter of the central nervous system, believed to be influenced by genetic and environmental factors, though the precise mechanisms remain unclear [135]. EAE is a widely used model for studying MS [136]. Genome-wide association studies (GWAS) have identified BATF as a risk locus significantly associated with the mean upper cervical cord area (MUCCA) in MS patients. This association is related to the degree of atrophy and disability progression, suggesting that BATF is a potential prognostic marker for MS-related disability progression [137].

BATF plays a crucial role in regulating Th17 cell differentiation and significantly influences the development of EAE. Studies have demonstrated that BATF-deficient mice immunized with MOG35-55 do not produce IL-17, thereby exhibiting resistance to EAE [4]. Additionally, the glycolytic intermediate phosphoenolpyruvate (PEP) can bind to JunB, inhibiting the JunB/BATF/IRF4 complex from binding to the IL-17 and RORγT gene loci, thereby suppressing Th17 cell differentiation and IL-17 production, alleviating Th17-dependent autoimmune encephalomyelitis [13]. Furthermore, the 35 kDa interferon-induced protein (IFP35) is highly expressed in MS patients, which is significant for human central nervous system demyelinating diseases [138]. IFP35 is an interferon-stimulated gene (ISG) that forms a homodimer with N-myc-interactor (NMI) and BATF, stabilizing the protein structure of IFP35 and facilitating its nuclear translocation and subsequent functional activation [138, 139] (Fig. 3; Table 2).

In summary, BATF influences the development and progression of MS by affecting the function of various immune cell types. Thus, modulating the expression or activity of BATF may serve as a strategy for treating MS, including the use of drugs to regulate BATF’s expression or activity, modulating immune cell responses, and alleviating inflammation and immune-mediated damage.

### Experimental autoimmune uveitis

EAU is an organ-specific autoimmune disease primarily mediated by T cells, commonly used as a model to study uveitis [140]. BATF is implicated in the progression of EAU. In IL-27-treated EAU mice, IL-27 enhances the binding of BATF to IRF8, promoting the differentiation and development of IL-27 regulatory B cells (Breg), while reducing the populations of Th1 and Th17 cells [141]. Similarly, in IL-35-treated EAU mice, IL-35 facilitates the binding of BATF to IRF4, leading to the differentiation and development of IL-35 Breg cells, which secrete IL-35 and IL-10 cytokines, thereby alleviating EAU [142]. The mechanism involves the recruitment of BATF, IRF4, and IRF8 to the AICE regions of the IL-12 A and EBI3 gene loci, promoting the secretion of IL-35 by Breg cells and subsequently mitigating EAU [143] (Fig. 3; Table 2).

Thus, targeting the key transcription factor BATF in Breg cells may be an effective strategy for the treatment of EAU.

### Rheumatoid arthritis

RA is a systemic autoimmune disease characterized by recurrent symmetric inflammation of the joints, including the hands, wrists, feet, and knees [144]. Collagen-induced arthritis (CIA) is a well-established rodent model that closely resembles human RA [145]. In the CIA mouse model, BATF levels are significantly elevated in the inflammatory synovial tissue [11]. Studies indicate that BATF-deficient mice exhibit reduced production of autoantibodies and a marked delay in CIA development [11]. Furthermore, BATF deficiency also mitigates symptoms such as synovitis, synovial hyperplasia, and angiogenesis in the synovial tissue [11]. These mice show decreased expression of RANKL and reduced osteoclast differentiation, which helps prevent bone erosion [11]. BATF exacerbates CIA by regulating Th17 cell differentiation and promoting the conversion of Treg cells into Th17 cells [11]. Research also shows that the absence of the immediate early response gene X-1 (IEX-1) increases mitochondrial reactive oxygen species (ROS) production following T cell activation, leading to elevated BATF expression, enhanced Th17 cell differentiation, and more severe arthritis [146]. Additionally, IL-18 and IL-23 can induce neutrophils to produce IL-17 A, IL-17 F, and IL-22 via the JNK/p38-STAT3-BATF pathway, thereby promoting the onset of CIA [147]. In RA patients, BATF is upregulated in eTreg cells within the inflammatory exudate [148, 149]. Moreover, fibroblast-like synoviocytes (RA FLS) in RA show high BATF expression, though the precise mechanisms remain to be elucidated [150]. Overexpression of BATF may contribute to synovial inflammation [150] (Fig. 3; Table 2).

In summary, BATF plays a role in regulating immune and stromal cells in CIA, suggesting that BATF could be a potential therapeutic target for treating RA. This discovery provides a significant biological basis for the development of targeted drugs to alleviate RA.

### Systemic lupus erythematosus

SLE is a chronic autoimmune disease affecting multiple organs, characterized primarily by autoantibody production and immune complex deposition [151]. Genome-wide association studies have identified overexpression of the transcription factor BATF in the promoters of susceptibility genes in SLE patients [152]. Elevated levels of BATF2 have also been observed in CD4^+^T cells from lupus patients [153]. In spontaneous lupus mouse models, BATF protein and RNA levels are significantly increased, particularly in the kidneys, where BATF expression positively correlates with RORγT and IL-17 levels [154]. Egr-2 has been shown to bind to BATF and inhibit its activity by reducing its association with AP-1 family members, thus impeding Th17 cell differentiation and exacerbating related diseases [155, 156]. In SLE patients, targeting Rho-associated kinase 2 (ROCK2) reduces BATF expression in Tfh cells, thereby decreasing Tfh cell generation and slowing disease progression [157]. OX40 signaling induces BATF upregulation in primary CD4^+^T cells, which enhances Tfh cell involvement and accelerates SLE progression [158]. Treatment of MRL/lpr mice with the traditional Chinese medicine formula “Langchuangding” results in decreased BATF and RORγT expression, effectively alleviating disease symptoms [159]. Beyond the commonly studied Tfh and Th17 cells, Th2 and Th9 cells also contribute to SLE pathogenesis. BATF promotes Th2 cell production by binding to RHF6 and RHS7 [160, 161]. In Th9 cells, BATF expression is significantly upregulated in SLE patients, and inhibition of the SPI1 gene can mitigate disease progression by targeting BATF in Th9 cells [162]. Chromatin accessibility analysis (ATAC-seq) in SLE patients shows that BATF expression is not only higher compared to healthy individuals but also exhibits increased chromatin accessibility, suggesting additional functions for BATF in SLE B cells [163]. Furthermore, treatment with the glucocorticoid RCI inhibits both AICDA and BATF, suppressing B cell function [164]. Non-classical memory B cells (ATM) in active lupus patients show increased BATF expression and contribute to disease progression [165]. Additionally, double deficiency of the DEF6 and SWAP-70 genes results in excessive accumulation of age-related B cells and spontaneous lupus. w Transcriptome and chromatin analysis reveal enriched chromatin open regions for molecules like IRF, BATF, and T-bet, facilitating their transcription [166]. Thus, the roles of BATF in SLE progression warrant further investigation (Fig. 3; Table 2).

### Other autoimmune diseases

Pemphigus is an autoimmune disease characterized by the formation of antibody-mediated blisters on the skin and mucous membranes. The condition is primarily triggered by autoantibodies against desmoglein (DSG) [167]. Research has identified autoreactive B cells as crucial in the pathogenesis of pemphigus, with the transcription factor BATF being highly expressed in DSG-specific B cells, suggesting its potential as a diagnostic marker [168]. Another autoimmune disorder, immune-related pancytopenia (IRP), is marked by the production of autoantibodies against platelets [169]. In IRP, Th9 cells may contribute to autoantibody production by B cells through the secretion of IL-9, which can trigger the disease [169]. Patients with IRP exhibit an increase in peripheral blood Th9 cells and elevated serum IL-9 levels, accompanied by a significant increase in BATF expression, which is associated with Th9 cells [169]. Additionally, GWAS have demonstrated that BATF is highly enriched in susceptibility genes for type 1 diabetes (T1D) [170]. In summary, BATF plays a critical role in the pathogenesis of these autoimmune diseases and may serve as a potential therapeutic target by inhibiting the activation of autoreactive cells to mitigate disease progression (Fig. 3; Table 2).

**Table 1:** Role of BATF in immune cell populations

Cell type	Function	Ref
ILCs	BATF deficiency results in a decrease in ILC lymphocytes, resulting in a defective ILC response to inflammatory cytokines and immunodeficiency to intestinal bacterial infections.	[25, 61]
DCs	BATF3-deficient mice exhibit impaired antigen cross-presentation, impaired CTL response to viral infection, and impaired response to tumor attack.	[53, 70]
Th17 cells	BATF binds to JunB and IRF4 as BATF/JUNB/IRF4 trimers and functions as a precursor to Th17 nuclear transcription factor binding during cytokine-stimulated differentiation.	[4, 14]
Tfh cells	BATF works synergistically with IRF4, STAT3, and STAT6 to activate IL-4 production in Tfh cells by directly binding to and activating the CNS2 region of the IL-4 gene. And regulates the expression of Bcl6 and c-Maf, important factors for Tfh cell differentiation.	[30, 164]
Th2 cells	BATF-deficient mice have a reduced ability to produce Th2-related cytokines, thereby promoting allergic inflammation.	[88]
Th9 cells	BATF binds to conserved regulatory elements of the IL9 gene, thereby stabilizing IL-9 expression and supporting the differentiation of Th9 cells.	[28, 171]
Treg cells	BATF is a key regulator of tissue Treg cells and suggests that sequence-specific perturbations of Foxp3-DNA interactions can affect specific aspects of Treg cell physiology and the immunopathology it regulates.	[99, 103]
CD8^+^T cells	BATF is a central regulator of early-effector CD8^+^T cell differentiation. CD8^+^T cells lacking BATF are unable to perform normal naïve differentiation and proliferative proliferation. BATF is responsible for balancing the immune status of T cells.	[7, 115]
B cells	BATF plays an important role in antigen-triggered germinal center formation and class-switching antibody production.	[46, 127]

**Table 2:** BATF-related autoimmune diseases

Disease	Relevant cells	Function	Ref	
IBD	Th1/Th17/Tregcells	Promotes the proliferation and differentiation of Th17 cells and interacts with IRF4 to produce cytokines such as IL-17 A. Activates local tissue Foxp3^+^Treg cells.	[12, 135, 172]	
MS	Th17/Tfh cells	The formation of BATF/Jun/IRF4 complex promotes the differentiation of Th17 and Tfh cells, and produces cytokines such as IL-21, which play a role in disease progression.	[4, 13]	
EAU	Th1/Th17 /Breg cells	BATF promotes the differentiation and development of IL-27Breg and IL-35Breg cells by interacting with IRF8 and IRF4, enhancing its protective effect against EAU.	[141, 143]	
SLE	Th17/Tfh /Th2/Th9/B cells	BATF plays a role in SLE by regulating the differentiation and function of Th17, Tfh, Th2, and Th9 cell subsets, promoting inflammatory responses and humoral immune reactions. In B cells, BATF is involved in regulating antibody production and the formation of memory B cells.	[157, 159, 165]	
RA	Th17 cells	BATF exacerbates the symptoms of CIA by regulating the differentiation of Th17 cells and the conversion of Treg cells to Th17 cells. Additionally, the absence of IEX-1 increases mitochondrial reactive oxygen species production, and the IL-18 and IL-23-induced JNK/p38-STAT3-BATF pathway promotes inflammation.	[146, 147]	
Pemphigus /IRP	Th9/B cells	BATF promotes the differentiation of Th9 cells. In B cells, BATF regulates gene expression, development, and antibody production, influencing the effectiveness and persistence of humoral immune responses.	[168, 169]	

## Conclusions

This review summarizes the research progress in elucidating the roles of BATF in regulating immune cell differentiation and function, with a particular focus on its implication in the development of autoimmune diseases. Both the functions and regulatory mechanisms of BATF exhibit complex characteristics in the pathogenesis of autoimmune diseases, exhibiting a key role in disease onset and progression by regulating immune cell function, immune tolerance, and inflammatory responses. Current studies on how BATF specifically regulates autoimmune diseases is mainly focused on molecular mechanisms and has not yet been widely applied in clinical trials. Additionally, the expression levels of BATF and its family members are associated with autoimmune diseases, suggesting that they may serve as potential disease biomarkers or diagnostic tools to aid for early diagnosis and prognosis evaluation. However, the exact mechanisms of BATF in autoimmune diseases require further investigation for a deeper understanding of autoimmune pathogenesis, which may contribute to the development of small interfering RNA or small molecule drugs targeting BATF for clinical treatment of various autoimmune diseases.

## Data Availability

No datasets were generated or analysed during the current study.

## References

[1] Sopel N, et al. The transcription factor BATF modulates cytokine-mediated responses in T cells. Cytokine Growth Factor Rev. 2016;30:39–45. 26970726 10.1016/j.cytogfr.2016.03.004

[2] Angel P, Karin M. The role of Jun, Fos and the AP-1 complex in cell-proliferation and transformation. Biochim Biophys Acta. 1991;1072(2–3):129–57. 1751545 10.1016/0304-419x(91)90011-9

[3] Murphy TL, Tussiwand R, Murphy KM. Specificity through cooperation: BATF-IRF interactions control immune-regulatory networks. Nat Rev Immunol. 2013;13(7):499–509. 23787991 10.1038/nri3470

[4] Schraml BU, et al. The AP-1 transcription factor Batf controls T(H)17 differentiation. Nature. 2009;460(7253):405–9. 19578362 10.1038/nature08114 PMC2716014

[5] Sahoo A, et al. Batf is important for IL-4 expression in T follicular helper cells. Nat Commun. 2015;6:7997. 26278622 10.1038/ncomms8997 PMC4557271

[6] Betz BC, et al. Batf coordinates multiple aspects of B and T cell function required for normal antibody responses. J Exp Med. 2010;207(5):933–42. 20421391 10.1084/jem.20091548 PMC2867277

[7] Kurachi M, et al. The transcription factor BATF operates as an essential differentiation checkpoint in early effector CD8 + T cells. Nat Immunol. 2014;15(4):373–83. 24584090 10.1038/ni.2834 PMC4000237

[8] Zhang X, et al. Depletion of BATF in CAR-T cells enhances antitumor activity by inducing resistance against exhaustion and formation of central memory cells. Cancer Cell. 2022;40(11):1407–e14227. 36240777 10.1016/j.ccell.2022.09.013

[9] Ubel C, et al. The activating protein 1 transcription factor basic leucine zipper transcription factor, ATF-like (BATF), regulates lymphocyte- and mast cell-driven immune responses in the setting of allergic asthma. J Allergy Clin Immunol. 2014;133(1):198–e2061. 24290279 10.1016/j.jaci.2013.09.049

[10] Hildner K, et al. Immunopathogenesis of IBD: Batf as a key driver of disease activity. Dig Dis. 2016;34(Suppl 1):40–7. 27548324 10.1159/000447281

[11] Park SH, et al. BATF regulates collagen-induced arthritis by regulating T helper cell differentiation. Arthritis Res Ther. 2018;20(1):161. 30071881 10.1186/s13075-018-1658-0 PMC6090970

[12] Liu B et al. An inducible circular RNA circKcnt2 inhibits ILC3 activation to facilitate colitis resolution. Nat Commun. 2020;11(1). 10.1038/s41467-020-17944-5 PMC7427797 32796851

[13] Huang TY, et al. Phosphoenolpyruvate regulates the Th17 transcriptional program and inhibits autoimmunity. Cell Rep. 2023;42(3):112205. 36857180 10.1016/j.celrep.2023.112205

[14] Ciofani M, et al. A validated regulatory network for Th17 cell specification. Cell. 2012;151(2):289–303. 23021777 10.1016/j.cell.2012.09.016 PMC3503487

[15] Chang YK, Zuo Z, Stormo GD. Quantitative profiling of BATF family proteins/JUNB/IRF hetero-trimers using Spec-Seq. BMC Mol Biol. 2018;19(1):5. 29587652 10.1186/s12867-018-0106-7 PMC5869772

[16] Ise W, et al. The transcription factor BATF controls the global regulators of class-switch recombination in both B cells and T cells. Nat Immunol. 2011;12(6):536–43. 21572431 10.1038/ni.2037 PMC3117275

[17] Johansen LM, et al. EBNA2 and activated notch induce expression of BATF. J Virol. 2003;77(10):6029–40. 12719594 10.1128/JVI.77.10.6029-6040.2003 PMC154003

[18] Dorsey MJ, et al. B-ATF: a novel human bZIP protein that associates with members of the AP-1 transcription factor family. Oncogene. 1995;11(11):2255–65. 8570175

[19] Giunco S, et al. Extra-telomeric functions of telomerase in the pathogenesis of Epstein-Barr virus-driven B-cell malignancies and potential therapeutic implications. Infect Agent Cancer. 2018;13:14. 29643934 10.1186/s13027-018-0186-5 PMC5892012

[20] Giunco S, et al. Cross talk between EBV and telomerase: the role of TERT and NOTCH2 in the switch of latent/lytic cycle of the virus. Cell Death Dis. 2015;6(5):e1774. 26018735 10.1038/cddis.2015.145 PMC4669716

[21] Meyer NP, et al. Genomic organization of human B-ATF, a target for regulation by EBV and HTLV-1. Mamm Genome. 1998;9(10):849–52. 9745044 10.1007/s003359900881

[22] Eferl R, Wagner EF. AP-1: a double-edged sword in tumorigenesis. Nat Rev Cancer. 2003;3(11):859–68. 14668816 10.1038/nrc1209

[23] Karin M, Liu Z, Zandi E. AP-1 function and regulation. Curr Opin Cell Biol. 1997;9(2):240–6. 9069263 10.1016/s0955-0674(97)80068-3

[24] Echlin DR, et al. B-ATF functions as a negative regulator of AP-1 mediated transcription and blocks cellular transformation by Ras and Fos. Oncogene. 2000;19(14):1752–63. 10777209 10.1038/sj.onc.1203491

[25] Liu Q et al. BATF regulates innate lymphoid cell hematopoiesis and homeostasis. Sci Immunol. 2020;5(54). 10.1126/sciimmunol.aaz8154 PMC8375455 33277375

[26] Liao J, et al. Batf promotes growth arrest and terminal differentiation of mouse myeloid leukemia cells. Mol Cancer Res. 2011;9(3):350–63. 21296860 10.1158/1541-7786.MCR-10-0375 PMC3060294

[27] Wang J, et al. A differentiation checkpoint limits hematopoietic stem cell self-renewal in response to DNA damage. Cell. 2012;148(5):1001–14. 22385964 10.1016/j.cell.2012.01.040

[28] Jabeen R, et al. Th9 cell development requires a BATF-regulated transcriptional network. J Clin Invest. 2013;123(11):4641–53. 24216482 10.1172/JCI69489 PMC3809790

[29] Tsuda M, et al. A role for BATF3 in T(H)9 differentiation and T-cell-driven mucosal pathologies. Mucosal Immunol. 2019;12(3):644–55. 30617301 10.1038/s41385-018-0122-4 PMC6462229

[30] Liu X, Nurieva RI, Dong C. Transcriptional regulation of follicular T-helper (tfh) cells. Immunol Rev. 2013;252(1):139–45. 23405901 10.1111/imr.12040 PMC3579502

[31] Li P, et al. Complex interactions of transcription factors in mediating cytokine biology in T cells. Immunol Rev. 2014;261(1):141–56. 25123282 10.1111/imr.12199 PMC4174316

[32] Zheng Y. Foxp3 mutant undermines Treg cell function. Immunity. 2017;47(2):211–4. 28813651 10.1016/j.immuni.2017.07.024

[33] Khatun A, et al. BATF is required for Treg Homeostasis and stability to prevent autoimmune pathology. Adv Sci (Weinh). 2023;10(28):e2206692. 37587835 10.1002/advs.202206692 PMC10558681

[34] Ataide MA, et al. BATF3 programs CD8(+) T cell memory. Nat Immunol. 2020;21(11):1397–407. 32989328 10.1038/s41590-020-0786-2

[35] Qiu Z, Edge C, et al. Batf3 expression by CD8 T cells critically regulates the development of memory populations. J Immunol. 2020;205(4):901–6. 32669309 10.4049/jimmunol.2000228 PMC7539233

[36] Roy S, et al. Batf2/Irf1 induces inflammatory responses in classically activated macrophages, lipopolysaccharides, and mycobacterial infection. J Immunol. 2015;194(12):6035–44. 25957166 10.4049/jimmunol.1402521

[37] Li C, et al. BATF2 balances the T cell-mediated immune response of CADM with an anti-MDA5 autoantibody. Biochem Biophys Res Commun. 2021;551:155–60. 33740622 10.1016/j.bbrc.2021.02.128

[38] van der Geest R, et al. BATF2 enhances proinflammatory cytokine responses in macrophages and improves early host defense against pulmonary Klebsiella pneumoniae infection. Am J Physiol Lung Cell Mol Physiol. 2023;325(5):L604–16. 37724373 10.1152/ajplung.00441.2022 PMC11068429

[39] Le DT, et al. BATF2 promotes HSC myeloid differentiation by amplifying IFN response mediators during chronic infection. iScience. 2023;26(2):106059. 36824275 10.1016/j.isci.2023.106059 PMC9942003

[40] Watowich SS, Liu YJ. Mechanisms regulating dendritic cell specification and development. Immunol Rev. 2010;238(1):76–92. 20969586 10.1111/j.1600-065X.2010.00949.x PMC3039024

[41] Grajales-Reyes GE, et al. Batf3 maintains autoactivation of Irf8 for commitment of a CD8α(+) conventional DC clonogenic progenitor. Nat Immunol. 2015;16(7):708–17. 26054719 10.1038/ni.3197 PMC4507574

[42] Taniguchi T, et al. IRF family of transcription factors as regulators of host defense. Annu Rev Immunol. 2001;19:623–55. 11244049 10.1146/annurev.immunol.19.1.623

[43] Paun A, Pitha PM. The IRF family, revisited. Biochimie. 2007;89(6–7):744–53. 17399883 10.1016/j.biochi.2007.01.014 PMC2139905

[44] Li P, et al. BATF-JUN is critical for IRF4-mediated transcription in T cells. Nature. 2012;490(7421):543–6. 22992523 10.1038/nature11530 PMC3537508

[45] Glasmacher E, et al. A genomic regulatory element that directs assembly and function of immune-specific AP-1-IRF complexes. Science. 2012;338(6109):975–80. 22983707 10.1126/science.1228309 PMC5789805

[46] Morman RE, et al. BATF regulates the expression of Nfil3, Wnt10a and miR155hg for efficient induction of antibody class switch recombination in mice. Eur J Immunol. 2018;48(9):1492–505. 29898247 10.1002/eji.201747360 PMC6357966

[47] Titcombe PJ et al. BATF represses BIM to sustain tolerant T cells in the periphery. J Exp Med. 2023;220(12). 10.1084/jem.20230183 PMC10588758 37862030

[48] Seo H, et al. IRF4 cooperate to counter exhaustion in tumor-infiltrating CAR T cells. Nat Immunol. 2021;22(8):983–95. 34282330 10.1038/s41590-021-00964-8 PMC8319109

[49] Deppmann CD, et al. Phosphorylation of BATF regulates DNA binding: a novel mechanism for AP-1 (activator protein-1) regulation. Biochem J. 2003;374(Pt 2):423–31. 12809553 10.1042/BJ20030455 PMC1223616

[50] Williams KL, et al. Characterization of murine BATF: a negative regulator of activator protein-1 activity in the thymus. Eur J Immunol. 2001;31(5):1620–7. 11466704 10.1002/1521-4141(200105)31:5<1620::aid-immu1620>3.0.co;2-3

[51] Deppmann CD, Taparowsky EJ. Reverse-polarity PAGE for examining DNA binding domain phosphorylation. Biotechniques. 2003;34(1):56–9. 12545539 10.2144/03341bm08

[52] Tussiwand R, et al. Compensatory dendritic cell development mediated by BATF-IRF interactions. Nature. 2012;490(7421):502–7. 22992524 10.1038/nature11531 PMC3482832

[53] Hildner K, et al. Batf3 deficiency reveals a critical role for CD8alpha + dendritic cells in cytotoxic T cell immunity. Science. 2008;322(5904):1097–100. 19008445 10.1126/science.1164206 PMC2756611

[54] Edelson BT, et al. Peripheral CD103 + dendritic cells form a unified subset developmentally related to CD8alpha + conventional dendritic cells. J Exp Med. 2010;207(4):823–36. 20351058 10.1084/jem.20091627 PMC2856032

[55] Li C, et al. BATF3 deficiency alters CD8 + effector/exhausted T cells balance in skin transplantation. Mol Med. 2024;30(1):16. 38297190 10.1186/s10020-024-00792-0 PMC10832090

[56] Iwata A, et al. Quality of TCR signaling determined by differential affinities of enhancers for the composite BATF-IRF4 transcription factor complex. Nat Immunol. 2017;18(5):563–72. 28346410 10.1038/ni.3714 PMC5401770

[57] Wang Y, et al. Genetically targeting the BATF family transcription factors BATF and BATF3 in the mouse abrogates effector T cell activities and enables long-term heart allograft survival. Am J Transpl. 2022;22(2):414–26. 10.1111/ajt.16861 PMC8813885 34599765

[58] Clottu AS, et al. Innate lymphoid cells in Autoimmune diseases. Front Immunol. 2021;12:789788. 35069567 10.3389/fimmu.2021.789788 PMC8777080

[59] Klose CSN, et al. Differentiation of type 1 ILCs from a common progenitor to all helper-like innate lymphoid cell lineages. Cell. 2014;157(2):340–56. 24725403 10.1016/j.cell.2014.03.030

[60] Ebbo M, et al. Innate lymphoid cells: major players in inflammatory diseases. Nat Rev Immunol. 2017;17(11):665–78. 28804130 10.1038/nri.2017.86

[61] Wu X, et al. BATF promotes group 2 innate lymphoid cell-mediated lung tissue protection during acute respiratory virus infection. Sci Immunol. 2022;7(67):eabc9934. 35030033 10.1126/sciimmunol.abc9934 PMC9005262

[62] Miller MM et al. BATF acts as an essential regulator of IL-25-responsive migratory ILC2 cell fate and function. Sci Immunol. 2020;5(43). 10.1126/sciimmunol.aay3994 PMC7112987 31924686

[63] van der Ploeg EK et al. Steroid-resistant human inflammatory ILC2s are marked by CD45RO and elevated in type 2 respiratory diseases. Sci Immunol. 2021;6(55). 10.1126/sciimmunol.abd3489 33514640

[64] Vivier E, et al. Innate lymphoid cells: 10 years on. Cell. 2018;174(5):1054–66. 30142344 10.1016/j.cell.2018.07.017

[65] Korchagina AA et al. Transcriptional control of ILC identity. Front Immunol. 2023;14. 10.3389/fimmu.2023.1146077 PMC10033543 36969171

[66] Waisman A, et al. Dendritic cells as gatekeepers of tolerance. Semin Immunopathol. 2017;39(2):153–63. 27456849 10.1007/s00281-016-0583-z

[67] Weber JK. Characterization of the impact of the transcription factor BATF on antigen cross-presentation of plasmacytoid dendritic cells. 2024.

[68] Ali S et al. BATF controls IFN I production via DC-SCRIPT in plasmacytoid dendritic cells. bioRxiv. 2024:2024.01.11.574638.

[69] Ashok D, et al. Cross-presenting dendritic cells are required for control of Leishmania major infection. Eur J Immunol. 2014;44(5):1422–32. 24643576 10.1002/eji.201344242

[70] Desai P et al. Batf3-Dependent dendritic cells promote optimal CD8 T cell responses against respiratory Poxvirus infection. J Virol. 2018;92(16). 10.1128/JVI.00495-18 PMC6069197 29875235

[71] Wu B, Wan Y. Molecular control of pathogenic Th17 cells in autoimmune diseases. Int Immunopharmacol. 2020;80:106187. 31931372 10.1016/j.intimp.2020.106187 PMC7031035

[72] Song X, Gao H, Qian Y. Th17 differentiation and their pro-inflammation function. Adv Exp Med Biol. 2014;841:99–151. 25261206 10.1007/978-94-017-9487-9_5

[73] Yasuda K, Takeuchi Y, Hirota K. The pathogenicity of Th17 cells in autoimmune diseases. Semin Immunopathol. 2019;41(3):283–97. 30891627 10.1007/s00281-019-00733-8

[74] Yang J, et al. Targeting Th17 cells in autoimmune diseases. Trends Pharmacol Sci. 2014;35(10):493–500. 25131183 10.1016/j.tips.2014.07.006

[75] Zhao X, et al. The interplay of transcription and genome topology programs T cell development and differentiation. J Immunol. 2022;209(12):2269–78. 36469845 10.4049/jimmunol.2200625 PMC9731349

[76] Pham D, et al. Batf pioneers the reorganization of chromatin in developing effector T cells via Ets1-dependent recruitment of Ctcf. Cell Rep. 2019;29(5):1203–e12207. 31665634 10.1016/j.celrep.2019.09.064 PMC7182170

[77] Martinez GJ, Dong C. BATF: bringing (in) another Th17-regulating factor. J Mol Cell Biol. 2009;1(2):66–8. 19726487 10.1093/jmcb/mjp016

[78] Carr TM, et al. JunB promotes Th17 cell identity and restrains alternative CD4(+) T-cell programs during inflammation. Nat Commun. 2017;8(1):301. 28824171 10.1038/s41467-017-00380-3 PMC5563507

[79] Pham D, et al. Batf stabilizes Th17 cell development via impaired Stat5 recruitment of Ets1-Runx1 complexes. EMBO J. 2023;42(8):e109803. 36917143 10.15252/embj.2021109803 PMC10106990

[80] Smith PM, et al. IRAK-2 regulates IL-1-mediated pathogenic Th17 cell development in helminthic infection. PLoS Pathog. 2011;7(10):e1002272. 21998578 10.1371/journal.ppat.1002272 PMC3188523

[81] Shin B, et al. Mitochondrial oxidative phosphorylation regulates the fate decision between pathogenic Th17 and regulatory T cells. Cell Rep. 2020;30(6):1898–909. e4. 32049019 10.1016/j.celrep.2020.01.022 PMC9059282

[82] Zhu J, Helper Cell T. Differentiation, heterogeneity, and plasticity. Cold Spring Harb Perspect Biol. 2018;10(10). 10.1101/cshperspect.a030338 PMC6169815 28847903

[83] Wei X, Niu X. T follicular helper cells in autoimmune diseases. J Autoimmun. 2023;134. 10.1016/j.jaut.2022.102976 36525939

[84] Ellyard JI, Vinuesa CG. A BATF-ling connection between B cells and follicular helper T cells. Nat Immunol. 2011;12(6):519–20. 21587310 10.1038/ni.2042

[85] Mathew R, et al. A negative feedback loop mediated by the Bcl6-cullin 3 complex limits tfh cell differentiation. J Exp Med. 2014;211(6):1137–51. 24863065 10.1084/jem.20132267 PMC4042651

[86] Vaeth M, et al. Store-operated ca 2 + entry in follicular T cells controls humoral immune responses and autoimmunity. Immunity. 2016;44(6):1350–64. 27261277 10.1016/j.immuni.2016.04.013 PMC4917422

[87] Tang T, et al. Roles of BATF/JUN/IRF4 complex in tacrolimus mediated immunosuppression on tfh cells in acute rejection after liver transplantation. J Cell Physiol. 2021;236(3):1776–86. 32749698 10.1002/jcp.29953

[88] Bao K, et al. BATF modulates the Th2 Locus Control Region and regulates CD4 + T cell fate during antihelminth immunity. J Immunol. 2016;197(11):4371–81. 27798167 10.4049/jimmunol.1601371 PMC5123670

[89] Kuwahara M et al. Bach2–Batf interactions control Th2-type immune response by regulating the IL-4 amplification loop. Nat Commun. 2016;7(1). 10.1038/ncomms12596 PMC5025763 27581382

[90] Yamashita M, Kuwahara M. The critical role of Bach2 in regulating type 2 chronic airway inflammation. Int Immunol. 2018;30(9):397–402. 29529253 10.1093/intimm/dxy020

[91] Koch S, Sopel N, Finotto S. Th9 and other IL-9-producing cells in allergic asthma. Semin Immunopathol. 2017;39(1):55–68. 27858144 10.1007/s00281-016-0601-1

[92] Humblin E et al. IRF8-dependent molecular complexes control the Th9 transcriptional program. Nat Commun, 2017. 8(1): p. 2085. 10.1038/s41467-017-01070-w PMC5727025 29233972

[93] Jabeen R. Retroviral transduction and reporter assay: transcription factor cooperation in Th9 cell development. Methods Mol Biol. 2017;1585:155–66. 28477194 10.1007/978-1-4939-6877-0_12

[94] Takami M, et al. Cutting edge: AhR is a molecular target of calcitriol in human T cells. J Immunol. 2015;195(6):2520–3. 26276877 10.4049/jimmunol.1500344 PMC4561210

[95] Karim AF, et al. Toll like receptor 2 engagement on CD4(+) T cells promotes TH9 differentiation and function. Eur J Immunol. 2017;47(9):1513–24. 28665005 10.1002/eji.201646846 PMC5606324

[96] Hu B, et al. Transcription factor networks in aged naive CD4 T cells bias lineage differentiation. Aging Cell. 2019;18(4):e12957. 31264370 10.1111/acel.12957 PMC6612640

[97] Shimizu J, Yamazaki S, Sakaguchi S. Induction of tumor immunity by removing CD25 + CD4 + T cells: a common basis between tumor immunity and autoimmunity. J Immunol. 1999;163(10):5211–8. 10553041

[98] Yang K. Regulation of Treg cell metabolism and function in non-lymphoid tissues. Front Immunol. 2022;13:909705. 35720275 10.3389/fimmu.2022.909705 PMC9200993

[99] Hayatsu N, et al. Analyses of a mutant Foxp3 Allele Reveal BATF as a critical transcription factor in the differentiation and accumulation of tissue regulatory T cells. Immunity. 2017;47(2):268–e2839. 28778586 10.1016/j.immuni.2017.07.008

[100] Li T, et al. Transcriptional switches in melanoma T cells: facilitating polarizing into regulatory T cells. Int Immunopharmacol. 2024;137:112484. 38885605 10.1016/j.intimp.2024.112484

[101] Delacher M, et al. Precursors for nonlymphoid-tissue treg cells reside in secondary lymphoid organs and are programmed by the transcription factor BATF. Immunity. 2020;52(2):295–e31211. 10.1016/j.immuni.2019.12.002 PMC7026712 31924477

[102] Vasanthakumar A, et al. The transcriptional regulators IRF4, BATF and IL-33 orchestrate development and maintenance of adipose tissue-resident regulatory T cells. Nat Immunol. 2015;16(3):276–85. 25599561 10.1038/ni.3085

[103] Itahashi K, et al. BATF epigenetically and transcriptionally controls the activation program of regulatory T cells in human tumors. Sci Immunol. 2022;7(76):eabk0957. 36206353 10.1126/sciimmunol.abk0957

[104] Lee W, et al. The transcription factor Batf3 inhibits the differentiation of regulatory T cells in the periphery. Exp Mol Med. 2017;49(11):e393. 29147008 10.1038/emm.2017.157 PMC5704186

[105] Arnold PR, et al. Suppression of FOXP3 expression by the AP-1 family transcription factor BATF3 requires partnering with IRF4. Front Immunol. 2022;13:966364. 36090981 10.3389/fimmu.2022.966364 PMC9452699

[106] Zhang X, et al. OX40 costimulation inhibits foxp3 expression and Treg induction via BATF3-dependent and independent mechanisms. Cell Rep. 2018;24(3):607–18. 30021159 10.1016/j.celrep.2018.06.052 PMC6095196

[107] Godec J, et al. Inducible RNAi in vivo reveals that the transcription factor BATF is required to initiate but not maintain CD8 + T-cell effector differentiation. Proc Natl Acad Sci U S A. 2015;112(2):512–7. 25548173 10.1073/pnas.1413291112 PMC4299213

[108] Man K, et al. The transcription factor IRF4 is essential for TCR affinity-mediated metabolic programming and clonal expansion of T cells. Nat Immunol. 2013;14(11):1155–65. 24056747 10.1038/ni.2710

[109] Quigley M, et al. Transcriptional analysis of HIV-specific CD8 + T cells shows that PD-1 inhibits T cell function by upregulating BATF. Nat Med. 2010;16(10):1147–51. 20890291 10.1038/nm.2232 PMC3326577

[110] Kuroda S, et al. Basic leucine zipper transcription factor, ATF-like (BATF) regulates epigenetically and energetically effector CD8 T-cell differentiation via Sirt1 expression. Proc Natl Acad Sci U S A. 2011;108(36):14885–9. 21873234 10.1073/pnas.1105133108 PMC3169148

[111] Xin G, et al. Critical role of IL-21-induced BATF in sustaining CD8-T-cell-mediated chronic viral control. Cell Rep. 2015;13(6):1118–24. 26527008 10.1016/j.celrep.2015.09.069 PMC4859432

[112] Battegay M, et al. Enhanced establishment of a virus carrier state in adult CD4 + T-cell-deficient mice. J Virol. 1994;68(7):4700–4. 7911534 10.1128/jvi.68.7.4700-4704.1994 PMC236402

[113] Elsaesser H, Sauer K, Brooks DG. IL-21 is required to control chronic viral infection. Science. 2009;324(5934):1569–72. 19423777 10.1126/science.1174182 PMC2830017

[114] Kim DH, Kim HY, Lee WW. Induction of unique STAT heterodimers by IL-21 provokes IL-1RI expression on CD8(+) T cells, resulting in enhanced IL-1beta dependent effector function. Immune Netw. 2021;21(5):e33. 34796037 10.4110/in.2021.21.e33 PMC8568912

[115] Chen Y, et al. BATF regulates progenitor to cytolytic effector CD8(+) T cell transition during chronic viral infection. Nat Immunol. 2021;22(8):996–1007. 34282329 10.1038/s41590-021-00965-7 PMC9258987

[116] Li S, et al. Ablation of BATF alleviates transplant rejection via abrogating the effector differentiation and memory responses of CD8(+) T cells. Front Immunol. 2022;13:882721. 35514970 10.3389/fimmu.2022.882721 PMC9062028

[117] BATF and IRF4 prevent CAR T-cell exhaustion. Cancer Discov. 2021;11(9):Of9. 10.1158/2159-8290.CD-RW2021-109 34330783

[118] Man K, et al. Transcription factor IRF4 promotes CD8(+) T cell exhaustion and limits the development of memory-like T cells during chronic infection. Immunity. 2017;47(6):1129–e11415. 29246443 10.1016/j.immuni.2017.11.021

[119] Liu Q, et al. Potentially mediates negative regulation of PD-1/PD-Ls pathway on T cell functions in Mycobacterium tuberculosis infection. Front Immunol. 2019;10:2430. 31681314 10.3389/fimmu.2019.02430 PMC6803382

[120] Kagoya Y, et al. BET bromodomain inhibition enhances T cell persistence and function in adoptive immunotherapy models. J Clin Invest. 2016;126(9):3479–94. 27548527 10.1172/JCI86437 PMC5004946

[121] Ford BR, et al. Tumor microenvironmental signals reshape chromatin landscapes to limit the functional potential of exhausted T cells. Sci Immunol. 2022;7(74):eabj9123. 35930654 10.1126/sciimmunol.abj9123 PMC9851604

[122] McCutcheon SR, et al. Transcriptional and epigenetic regulators of human CD8(+) T cell function identified through orthogonal CRISPR screens. Nat Genet. 2023;55(12):2211–23. 37945901 10.1038/s41588-023-01554-0 PMC10703699

[123] Wei X, Niu X. T follicular helper cells in autoimmune diseases. J Autoimmun. 2023;134:102976. 36525939 10.1016/j.jaut.2022.102976

[124] Kang N, et al. Aberrant B-cell activation in systemic Lupus Erythematosus. Kidney Dis (Basel). 2022;8(6):437–45. 36590680 10.1159/000527213 PMC9798842

[125] Du W, et al. The multiple roles of B cells in the pathogenesis of Sjögren’s syndrome. Front Immunol. 2021;12:684999. 34168653 10.3389/fimmu.2021.684999 PMC8217880

[126] Xiao F, et al. Epigenetic regulation of B cells and its role in autoimmune pathogenesis. Cell Mol Immunol. 2022;19(11):1215–34. 36220996 10.1038/s41423-022-00933-7 PMC9622816

[127] Ochiai K, et al. Transcriptional regulation of germinal center B and plasma cell fates by dynamical control of IRF4. Immunity. 2013;38(5):918–29. 23684984 10.1016/j.immuni.2013.04.009 PMC3690549

[128] Lio CJ et al. TET enzymes augment activation-induced deaminase (AID) expression via 5-hydroxymethylcytosine modifications at the Aicda superenhancer. Sci Immunol. 2019;4(34). 10.1126/sciimmunol.aau7523 PMC6599614 31028100

[129] Lee SH, Kwon JE, Cho ML. Immunological pathogenesis of inflammatory bowel disease. Intest Res. 2018;16(1):26–42. 29422795 10.5217/ir.2018.16.1.26 PMC5797268

[130] Punkenburg E, et al. Batf-dependent Th17 cells critically regulate IL-23 driven colitis-associated colon cancer. Gut. 2016;65(7):1139–50. 25838550 10.1136/gutjnl-2014-308227

[131] Li Y, et al. Characterization and biological significance of IL-23-induced neutrophil polarization. Cell Mol Immunol. 2018;15(5):518–30. 28690333 10.1038/cmi.2017.39 PMC6068162

[132] Devlin JC, et al. Single-cell transcriptional survey of ileal-anal pouch immune cells from ulcerative colitis patients. Gastroenterology. 2021;160(5):1679–93. 33359089 10.1053/j.gastro.2020.12.030 PMC8327835

[133] Wang C, et al. BATF is required for normal expression of gut-homing receptors by T helper cells in response to retinoic acid. J Exp Med. 2013;210(3):475–89. 23460729 10.1084/jem.20121088 PMC3600908

[134] Zeng B, et al. ILC3 function as a double-edged sword in inflammatory bowel diseases. Cell Death Dis. 2019;10(4):315. 30962426 10.1038/s41419-019-1540-2 PMC6453898

[135] Garg N, Smith TW. An update on immunopathogenesis, diagnosis, and treatment of multiple sclerosis. Brain Behav. 2015;5(9):e00362. 26445701 10.1002/brb3.362 PMC4589809

[136] Constantinescu CS, et al. Experimental autoimmune encephalomyelitis (EAE) as a model for multiple sclerosis (MS). Br J Pharmacol. 2011;164(4):1079–106. 21371012 10.1111/j.1476-5381.2011.01302.x PMC3229753

[137] Akkad DA, et al. Multiple sclerosis risk loci correlate with cervical cord atrophy and may explain the course of disability. Neurogenetics. 2015;16(3):161–8. 25620546 10.1007/s10048-015-0438-0

[138] De Masi R et al. IFP35 is a relevant factor in Innate Immunity, multiple sclerosis, and other Chronic Inflammatory diseases: a review. Biology (Basel). 2021;10(12). 10.3390/biology10121325 PMC8698480 34943240

[139] Wang X, et al. IFP 35 forms complexes with B-ATF, a member of the AP1 family of transcription factors. Biochem Biophys Res Commun. 1996;229(1):316–22. 8954125 10.1006/bbrc.1996.1799

[140] Kerr EC, et al. The dynamics of leukocyte infiltration in experimental autoimmune uveoretinitis. Prog Retin Eye Res. 2008;27(5):527–35. 18723108 10.1016/j.preteyeres.2008.07.001

[141] Choi JK et al. IL-27-producing B-1a cells suppress neuroinflammation and CNS autoimmune diseases. Proc Natl Acad Sci U S A. 2021;118(47). 10.1073/pnas.2109548118 PMC8617509 34782464

[142] Wang RX, et al. Interleukin-35 induces regulatory B cells that suppress autoimmune disease. Nat Med. 2014;20(6):633–41. 24743305 10.1038/nm.3554 PMC4048323

[143] Yu CR, et al. Production of IL-35 by Bregs is mediated through binding of BATF-IRF-4-IRF-8 complex to il12a and ebi3 promoter elements. J Leukoc Biol. 2018;104(6):1147–57. 30117603 10.1002/JLB.3A0218-071RRR PMC11290588

[144] Wang S, et al. Advances in experimental models of rheumatoid arthritis. Eur J Immunol. 2023;53(1):e2249962. 36330559 10.1002/eji.202249962

[145] Leung PS, et al. Development and validation of gene therapies in autoimmune diseases: epidemiology to animal models. Autoimmun Rev. 2010;9(5):A400–5. 20035901 10.1016/j.autrev.2009.12.009

[146] Zhi L, et al. Enhanced Th17 differentiation and aggravated arthritis in IEX-1-deficient mice by mitochondrial reactive oxygen species-mediated signaling. J Immunol. 2012;189(4):1639–47. 22798682 10.4049/jimmunol.1200528 PMC3440304

[147] Chen Y, et al. The effects of adoptively transferred IL-23/IL-18-polarized neutrophils on tumor and collagen-induced arthritis in mice. J Inflamm Res. 2021;14:4669–86. 34557012 10.2147/JIR.S329528 PMC8453247

[148] Lutter L, et al. Human regulatory T cells locally differentiate and are functionally heterogeneous within the inflamed arthritic joint. Clin Transl Immunol. 2022;11(10):e1420. 10.1002/cti2.1420 PMC9525321 36204213

[149] Mijnheer G, et al. Conserved human effector Treg cell transcriptomic and epigenetic signature in arthritic joint inflammation. Nat Commun. 2021;12(1):2710. 33976194 10.1038/s41467-021-22975-7 PMC8113485

[150] Zerrouk N, et al. Identification of putative master regulators in rheumatoid arthritis synovial fibroblasts using gene expression data and network inference. Sci Rep. 2020;10(1):16236. 33004899 10.1038/s41598-020-73147-4 PMC7529794

[151] Tenbrock K, Rauen T. T cell dysregulation in SLE. Clin Immunol. 2022;239:109031. 35526790 10.1016/j.clim.2022.109031

[152] Dozmorov MG, Wren JD, Alarcon-Riquelme ME. Epigenomic elements enriched in the promoters of autoimmunity susceptibility genes. Epigenetics. 2014;9(2):276–85. 24213554 10.4161/epi.27021 PMC3962538

[153] Li Z, et al. Identifying key genes in CD4(+) T cells of systemic lupus erythematosus by integrated bioinformatics analysis. Front Genet. 2022;13:941221. 36046235 10.3389/fgene.2022.941221 PMC9420982

[154] Ji LS et al. Mechanism of follicular helper T cell differentiation regulated by transcription factors. J Immunol Res, 2020. 2020: p. 1826587. 10.1155/2020/1826587 PMC7387970 32766317

[155] Sumitomo S, et al. Egr2 and Egr3 are the unique regulators for systemic autoimmunity. Jakstat. 2013;2(2):e23952. 24058814 10.4161/jkst.23952 PMC3710327

[156] Miao T, et al. Early growth response gene-2 controls IL-17 expression and Th17 differentiation by negatively regulating Batf. J Immunol. 2013;190(1):58–65. 23203924 10.4049/jimmunol.1200868

[157] Weiss JM, et al. ROCK2 signaling is required to induce a subset of T follicular helper cells through opposing effects on STATs in autoimmune settings. Sci Signal. 2016;9(437):ra73. 27436361 10.1126/scisignal.aad8953 PMC5193229

[158] Jacquemin C, et al. OX40 ligand contributes to human lupus pathogenesis by promoting T follicular helper response. Immunity. 2015;42(6):1159–70. 26070486 10.1016/j.immuni.2015.05.012 PMC4570857

[159] Zhang XF, et al. The effects of Langchuangding on the Th17 cell-related transcription factors BATF and RORγt in MRL_1pr mice. Industrial and organizational psychology; 2020.

[160] Wang Z, Chang C, Lu Q. Epigenetics of CD4 + T cells in autoimmune diseases. Curr Opin Rheumatol. 2017;29(4):361–8. 28362657 10.1097/BOR.0000000000000393

[161] Pan Q, Walls AF, Pan Q. Editorial: Th2-associated immunity in the pathogenesis of systemic lupus erythematosus and rheumatoid arthritis. Front Immunol, 2022. 13: p. 975553. 10.3389/fimmu.2022.975553 PMC9301996 35874701

[162] Zhao M, et al. 3D genome alterations in T cells associated with disease activity of systemic lupus erythematosus. Ann Rheum Dis. 2023;82(2):226–34. 36690410 10.1136/ard-2022-222653 PMC9887402

[163] Scharer CD, et al. ATAC-seq on biobanked specimens defines a unique chromatin accessibility structure in naive SLE B cells. Sci Rep. 2016;6:27030. 27249108 10.1038/srep27030 PMC4888756

[164] Qiu H, et al. Transcriptional and epigenetic regulation of follicular T-helper cells and their role in autoimmunity. Autoimmunity. 2017;50(2):71–81. 28263097 10.1080/08916934.2017.1284821

[165] Wu C, et al. Lupus-associated atypical memory B cells are mTORC1-hyperactivated and functionally dysregulated. Ann Rheum Dis. 2019;78(8):1090–100. 31142473 10.1136/annrheumdis-2019-215039 PMC6691860

[166] Manni M, et al. Regulation of age-associated B cells by IRF5 in systemic autoimmunity. Nat Immunol. 2018;19(4):407–19. 29483597 10.1038/s41590-018-0056-8 PMC6095139

[167] Schmidt E, Kasperkiewicz M, Joly P. Pemphigus. Lancet. 2019;394(10201):882–94. 31498102 10.1016/S0140-6736(19)31778-7

[168] Egami S, et al. Desmoglein-specific B-cell-targeted single-cell analysis revealing unique gene regulation in patients with pemphigus. J Invest Dermatol. 2023;143(10):1919–28. e16. 36997112 10.1016/j.jid.2023.03.1661

[169] Shao Q et al. Th9 cells in peripheral blood increased in patients with immune-related pancytopenia. J Immunol Res, 2020. 2020: p. 6503539. 10.1155/2020/6503539 PMC7222599 32455141

[170] Burren OS, Guo H, Wallace C. VSEAMS: a pipeline for variant set enrichment analysis using summary GWAS data identifies IKZF3, BATF and ESRRA as key transcription factors in type 1 diabetes. Bioinformatics. 2014;30(23):3342–8. 25170024 10.1093/bioinformatics/btu571 PMC4296156

[171] Sundrud MS, Hogan SP. What’s old is new again: Batf transcription factors and Th9 cells. Mucosal Immunol. 2019;12(3):583–5. 30833634 10.1038/s41385-019-0155-3

[172] Zhao J, et al. Th17 cells in inflammatory bowel disease: cytokines, plasticity, and therapies. J Immunol Res. 2021;2021:8816041. 33553436 10.1155/2021/8816041 PMC7846404

